# The intestinal microbial metabolite nicotinamide n-oxide prevents herpes simplex encephalitis via activating mitophagy in microglia

**DOI:** 10.1080/19490976.2022.2096989

**Published:** 2022-07-06

**Authors:** Feng Li, Yiliang Wang, Xiaowei Song, Zhaoyang Wang, Jiaoyan Jia, Shurong Qing, Lianzhou Huang, Yuan Wang, Shuai Wang, Zhe Ren, Kai Zheng, Yifei Wang

**Affiliations:** a Institute of Biomedicine, College of Life Science and Technology, Jinan University, Guangzhou, Guangdong, China; b Infectious Diseases Institute, Guangzhou Eighth People’s Hospital, Guangdong, China; c Institute of Molecular Rhythm and Metabolism, Guangzhou University of Chinese Medicine, Guangzhou, Guangdong, China; d School of Pharmaceutical Sciences, Health Science Center, Shenzhen University, Shenzhen, Guangdong, China

**Keywords:** HSE, gut microbiota, microglia, nicotinamide n-oxide, mitophagy

## Abstract

Herpes simplex encephalitis (HSE), a complication of herpes simplex virus type I (HSV-1) infection causes neurological disorder or even death in immunocompromised adults and newborns. However, the intrinsic factors controlling the HSE outcome remain unclear. Here, we show that HSE mice exhibit gut microbiota dysbiosis and altered metabolite configuration and tryptophan-nicotinamide metabolism. HSV-1 neurotropic infection activated microglia, with changed immune properties and cell numbers, to stimulate antiviral immune response and contribute substantially to HSE. In addition, depletion of gut microbiota by oral antibiotics (ABX)-treatment triggered the hyper-activation of microglia, which in turn enhanced inflammatory immune response, and cytokine production, resulting in aggregated viral burden and HSE pathology. Furthermore, exogenous administration of nicotinamide n-oxide (NAMO), an oxidative product of nicotinamide derived from gut microbiota, to ABX-treated or untreated HSE mice significantly diminished microglia-mediated proinflammatory response and limited HSV-1 infection in CNS. Mechanistic study revealed that HSV-1 activates microglia by increasing mitochondrial damage via defective mitophagy, whereas microbial metabolite NAMO restores NAD+-dependent mitophagy to inhibit microglia activation and HSE progression. NAMO also prevented neuronal cell death triggered by HSV-1 infection or microglia-mediated microenvironmental toxicity. Finally, we show that NAMO is mainly generated by neomycin-sensitive bacteria, especially *Lactobacillus_gasseri* and *Lactobacillus_reuteri*. Together, these data demonstrate that gut microbial metabolites act as intrinsic restrictive factors against HSE progression via regulating mitophagy in microglia, implying further exploration of bacterial or nutritional approaches for treating neurotropic virus-related neurodegenerative diseases.

## Introduction

Herpes simplex virus type I (HSV-1) is a neurotropic virus that causes several diseases, including herpes simplex encephalitis (HSE), keratitis, oral or facial lesions (*Herpes labialis*), and skin lesions (*Herpes gladiatorum*)^1^. Neuronal infection of HSV-1 also implicates in the pathological development of neurodegenerative diseases, such as Alzheimer’s disease and Parkinson’s disease.^1,2^ Despite improvements in antiviral treatment, HSE is still associated with neurological disorders and high mortality, especially in immunocompromised adults and newborns. Fatal HSE mainly results from immune pathology rather than virus replication-induced damage and different outcomes in HSE tropism following HSV-1 infection depend on the innate immune response controlling HSV-1 early dissemination in the central nervous system (CNS).^3,4^ It has recently emerged that different cell types adapt different antiviral innate signals to restrict HSV-1 replication and prevent HSE, among which microglia plays a central role.^5–11^ For instance, neurons and astrocytes utilized RNA-sensing TLR3 pathway,^6–10^ whereas microglia stimulated type I IFN-mediated antiviral action and enabled innate sensing pathway of other cell types in DNA-sensing cGAG-STING-dependent manner.^11^ Importantly, microglia induced antiviral response when challenged with lower HSV-1 doses, whereas underwent cGAS-dependent apoptosis at high virus loads to limit excessive type I IFN production and the augment of HSE pathology.^12^ Besides, microglia proliferation at early stage of HSE requires the activation of the macrophage colony-stimulating factor (MCSF)/macrophage colony-stimulating factor 1 receptor (CSF1R) axis.^13^ However, the regulatory mechanisms limiting microglia over-activation are unclear, and the acquired environmental or genetic factors that balance the beneficial or detrimental immune responses in microglia and how this response restricts HSV-1 level and HSE progression remain poorly explored.

Increasing evidence has revealed that gut microbiome orchestrate optimal innate and adaptive immune response to control virus infection, such as influenza virus (IAV), Lymphocytic choriomeningitis virus (LCMV), and hepatitis B virus (HBV).^14^ Enhanced susceptibility of gut microbiota-depleted mice, the antibiotics (ABX)-treated mice or germ-free (GF) mice, to lethal virus infection was associated with decreased production of type I IFN and antiviral IFN-stimulated genes,^15,16^ or associated with reduced pro-inflammatory cytokines, such as TNF-α, IL-6, and IL-18.^17,18^ Alternatively, the diminished virus-specific T cells and B cells in ABX-treatment and GF mice also contributed to the increased virus burden of IAV, LCMV, and HBV.^19–21^ Specific microbial constituents, metabolites, or microbe-associated molecular patterns (MAMPs) interacted with host receptors to alter type I IFN signaling and to prime various immune cell subsets during systemic virus infection.^22,23^ Similar to other viruses, microbiota dysbiosis caused by oral ABX treatment also impaired antiviral T cell responses and enhanced lethal HSV-2 infection.^24^ However, a mechanistic understanding of the gut microbiota influencing antiviral responses in HSV-1 infection and HSE progression is lacking.

Here, we described the impact of the intestinal microbiome on HSV-1 infection and HSE progression. HSE mice exhibited gut microbiota dysbiosis and altered metabolite configuration, which were partly reversed by HSV-1 clinically therapeutic drug Acyclovir (ACV). Besides, HSV-1 neurotropic infection of oral ABX-treated mice resulted in exacerbated viral burden and HSE pathology, which correlated with the enhanced immune response and inflammatory cytokine production triggered by hyper-activated microglia in ABX-treated mice. Furthermore, exogenous administration of nicotinamide n-oxide (NAMO), a gut microbiota metabolite, to ABX-treated or untreated HSE mice significantly reduced virus production and attenuated neuroinflammation. HSV-1 infection caused mitochondrial dysfunction and impaired mitophagy to activate microglia, whereas NAMO inhibited type I IFN signaling and restored mitophagy in microglia to restrict HSE progression. NAMO also prevented neuronal cell death triggered by HSV-1 infection or micro-environmental neuronal toxic factors released by microglia. Finally, NAMO was mainly generated by neomycin-sensitive bacteria, *Lactobacillus_gasseri* and *Lactobacillus_reuteri*. Together, perturbations in the intestinal microbiome or reconstitution with NAMO can modulated appropriated microglial-mediated immune responses in HSE progression through a mitophagy-dependent axis.

## Results

### 1. HSV-1 infection causes intestinal microbiota dysbiosis

To test whether HSV-1 infection affected intestinal flora, the mouse HSE model was established with intranasal HSV-1 infection. Several indexes were measured, including neurological disease scores (jumpy, uncoordinated, hunched/lethargic, unresponsive/nomovement and eye swell/lesions) (Supplemental figure 1A and 1B), weight loss (Supplemental figure 1C), survival (Supplemental figure 1D) and the area of HSV-1 in brain (Supplemental figure 1E), all of which indicated the successfully established HSE models.

**Figure 1.**
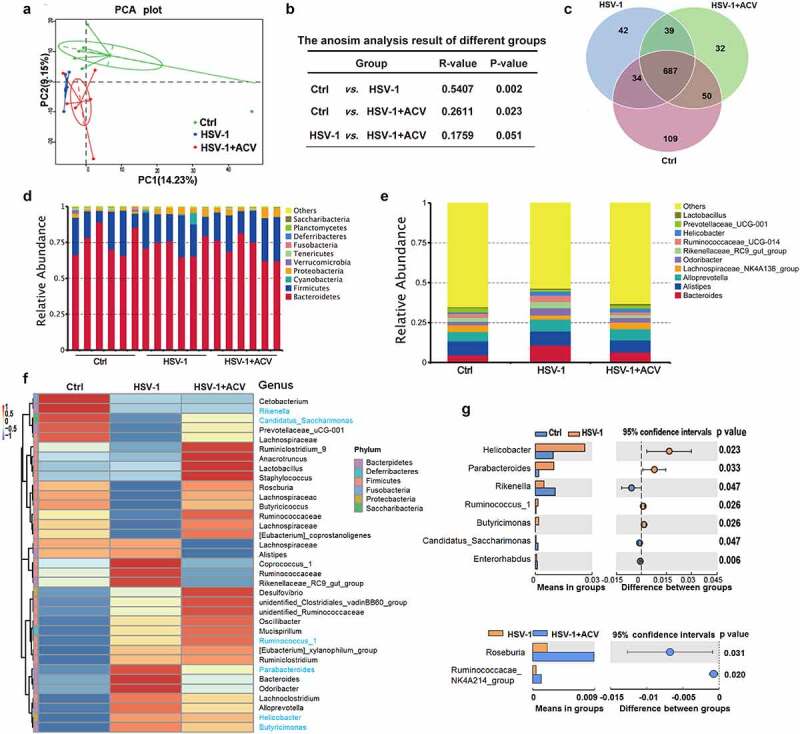
**Deregulation of intestinal flora in HSE mice**. (a) PCoA scatterplot showed clear clustering of gut bacterial communities in control (Ctrl), HSV-1, and HSV-1 + ACV groups. (b) Analysis of similarity (ANOSIM) showed microbial compositions. (c) Venn diagram showed the overlapped OTUs in Ctrl, HSV-1, and HSV-1 + ACV groups. (d-e) Top 10 microbes at the phylum level (d) or at the genus level (e) in Ctrl, HSV-1, and HSV-1 + ACV groups. (f) Heatmap for Top 35 microbes at the phylum level. Red indicated high expression and blue indicated low expression as shown in the scale bar. (g) Significant difference of bacteria in genus level between groups was analyzed by T-test.

The “gut-brain axis” incorporates bidirectional regulation of immune responses in the gut and brain, which is heavily influenced by intestinal microbes. We therefore investigated whether neuronal infection of HSV-1 affects gut microbes. Apparently, HSV-1 intranasal infection significantly promoted inflammation of the intestines by upregulating the cytokine production (Supplemental figure 1 F). Considering that the intestinal inflammation and gut microbiota are reciprocally regulated, the structural changes of intestinal flora upon HSV-1 infection were determined using 16s rRNA sequencing of microbes isolated from the feces of control mice (Ctrl), HSE mice (HSV-1) and HSE mice treated with ACV (HSV-1 + ACV). To characterize the levels and patterns of diversities within individuals, different measures of alpha diversity were performed (Supplemental figure 2A and 2B), which revealed no statistically significant differences in bacterial diversity and richness among the 3 groups. Besides, beta diversity of each group was calculated via the principal coordinates analysis (PCoA) based on unweighted UniFrac distances. As shown in Figure 1(a), the PCoA scatterplot indicated clear clustering of gut bacterial communities under HSV-1 infection and ACV treatment. Analysis of similarity (ANOSIM) further confirmed the significant separation of the groups (Ctrl vs HSV-1, R = 0.5407, p = .002) (Figure 1(b)), indicating clear differences in microbial compositions in the Ctrl and HSV-1 groups. Besides, all the groups shared a majority of their OTUs (Figure 1(c)). The top 10 microbes at the phylum and genus levels were shown, which also indicated the variations in the composition of the gut microbiota (Figure 1(d,e)). Analyses of the microbiota at the phylum level revealed a dominance of *Bacterioidetes* and *Firmicutes* in all the groups, and a great ratio of *Cyanobacteria* in HSV-1 group than that in Ctrl or HSV-1+ ACV group (Figure 1(d) and Supplemental figure 2C). Besides, HSV-1 infection upregulated the ratio of the genus *bacterioides, Odoribacter, Rikenellaceae_RC9_gut_group* and *Ruminococcaceae_UCG-014*, which were all reduced by ACV treatment (Figure 1(e,f) and Supplemental figure 2D). In addition, significant difference of bacteria in genus level between groups were analyzed by t-test, which demonstrated that HSV-1 infection dramatically enhanced the levels of *helicobacter* and *parabacteroides*, whereas ACV treatment restored the level of *Roseburia* (Figure 1(g)). Furthermore, HSV-1 infection upregulated the abundance of butyrate producing and SCFA-producing genera, suggesting the enhanced production of SCFA by gut microbiota (Supplemental figure 2E). Finally, the most different microbes at the species level were shown (Supplemental figure 2 F). HSV-1 infection significantly increased the abundance of *Bacteroides_sartorii, Bacteroides_acidifaciens*, and *Helicobacter*_*ganmani*, and conversely, reduced the ratio of *Lactobacillus*_*gasseri*, tendency of which was further reversed by ACV treatment. Together, the above results clearly revealed alterations in gut microbiota compositions in HSE.

### 2. Depletion of gut microbe aggregates HSE

**Figure 2.**
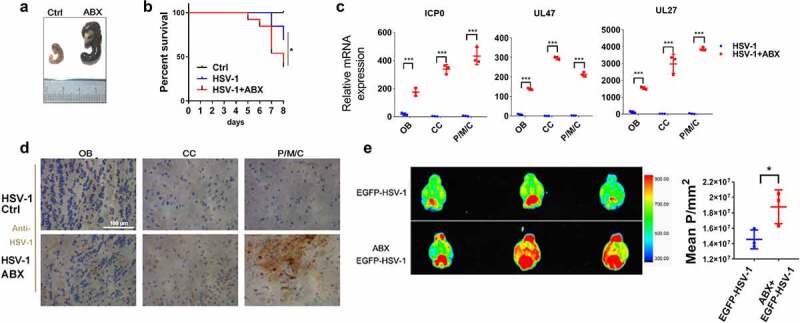
**Depletion of gut microbe aggravates HSE**. (a) Representative pictures of ceca from antibiotic-treated (ABX) and ctrl mice, with ruler for scaling. (b) Survival rates of Ctrl and HSV-1 infected normal or ABX-treated mice (log-rank test). n = 8–10 mice per group. (c) The mRNA expression levels of viral gene *ICP0, UL47* and *UL27* in olfactory bulb (OB), cerebral cortex (CC), and pons/medulla oblongata/cerebellum (P/M/C) derived from HSV-1 infected normal or ABX-treated mice. (d) Immunohistochemistry assay was performed to detect HSV-1 viral load in OB, CC, and P/M/C derived from HSV-1 infected normal or ABX-treated mice. Scale bar, 100 μm. (e) Normal or ABX-treated mice were infected with EGFP-HSV-1, and fluorescent images were captured by the Bruker Small Animal Optical Imaging System. Left panel: representative figures of EGFP-HSV-1 distribution in brain. Right panel: quantitative analysis of fluorescence intensity of EGFP-HSV-1. *p < .05, **p < .01 or ***p < .001 versus HSV-1 group.

To evaluate the role of gut microbe in HSE, we firstly depleted the intestinal microbiota of mice by administrating antibiotics (ABX) (mixes of vancomycin, neomycin, metronidazole, and ampicillin). As expected, broad-spectrum ABX markedly enlarged the size of ceca, without influence on the relative organ indexes (Figure 2(a) and supplemental figure 3A). ABX-treated mice also demonstrated slightly weight loss (Supplemental figure 3B). We next examined the gut microbe between ABX treatment and normal mice using 16S RNA sequencing. PCoA and ANOSIM analysis indicated clear clustering of gut bacterial communities under ABX treatment (Supplemental figure 3C and 3D). Besides, ABX significantly reduced both the alpha and beta diversity and compositions of the gut microbes (Supplemental figure 3E). A total of 321 OTUs were mutual abundant in the Ctrl and ABX group (Supplemental figure 3F). The microbiome of ABX-treated mice had far higher sequences from the *Proteobacteria* phylum and the *Enterobacter* genus (Supplemental figure 3G and 3 H). Many genera, such as *Bacteroides, Alloprevotella, Alistipes*, and *Lachnospiraceae* were reduced in the gut microbiota associated with ABX treatment, whereas *Enterobacter* was enriched (Supplemental figure 3I). Furthermore, several bacterial species, such as *Bacteroides_sartorii, Bacteroides_acidifaciens*, and *Helicobacter*_*ganmani*, were significantly reduced by ABX treatment (Supplemental figure 3 J and 3 K). In sum, antibiotics obviously reduced the diversity and compositions of gut microbiota.

We then examined the effect of ABX-mediated depletion of gut microbiota on HSE, and found that ABX treatment significantly reduced the survival rate of HSE mice (Figure 2(b)). Besides, histopathological and qRT-PCR analysis of different brain areas, including Olfactory bulb (OB), Cerebral cortex (CC), and Pons/medulla oblongata/cerebellum (P/M/C), demonstrated that ABX markedly upregulated HSV-1 virus loads and gene expression (Figure 2(c,d)). Finally, we used EGFP-tagged HSV-1 strain to visualize the effect of ABX on HSV-1-induced HSE. By in vivo imaging system, we found that EGFP-HSV-1 was mainly distributed in the cerebellum in Ctrl group. On the contrary, depletion of gut microbe by ABX enlarged the distribution of EGFP-HSV-1, which was not restricted to the area around the cerebellum but occupied larger areas of the brain, with a much stronger fluorescence intensity (Figure 2(e)). We also detected the distribution of EGFP-HSV-1 in other organs, and found no difference between the Ctrl and ABX group (Supplemental figure 3 L). Collectively, these data suggest that a continuous contribution of intestinal microbes prevents the development of HSE.

### 3. Lack of microbes enhances microglia immune response.

**Figure 3.**
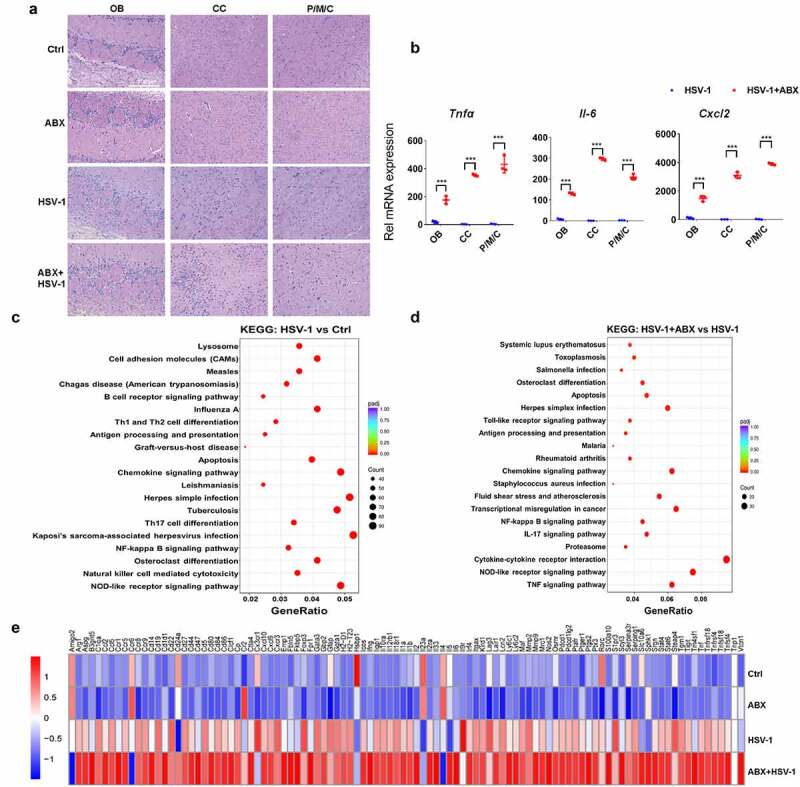
**Lack of microbes enhances immune response in mouse ob**. (a) H&E staining of OB, CC, and P/M/C tissue derived from Ctrl, ABX or ABX-treated mice infected with HSV-1. (b) total RNA of tissues derived from OB, CC, and P/M/C was extracted and the mRNA expression levels of *TNFα, IL-6* and *CXCL2* were analyzed by qRT-PCR, respectively. ***p < .001 versus HSV-1 group. (c-d) total RNA of OB tissue was extracted and the mRNA expression profile was detected by RNA-seq. Differentially expressed genes were analyzed by KEGG. The top signaling pathways identified as enriched in HSV-1-infected mice compared to mock-infected mice (c), or HSV-1-infected ABX-treated mice when compared to normal mice (d). (e) mRNA expression profile of representative inflammatory cytokines (at least 2-fold, p < .05) in OB from normal, ABX, HSV-1, and ABX + HSV-1 mice. Color code shows linear values.

Next, we examined whether depletion of intestinal bacteria influences the inflammatory response to HSV-1 infection. As revealed by histopathological and qRT-PCR analysis of OB, CC, P/M/C, AB pretreatment enhanced cellular damage and the expression of inflammatory cytokines upon HSV-1 infection (Figure 3(a,b)). To further investigate the impact of ABX on HSE, total RNA of OB in Ctrl, ABX-treated, HSV-1-infected, and ABX+HSV-1-treated group was extracted and the genome-wide mRNA expression profiles were measured by quantitative deep sequencing (RNA-seq). When compared the HSV-1 infected group with the Ctrl group, there were 2403 genes significantly upregulated by HSV-1 infection (Supplemental figure 4A), and according to the Gene Ontology (GO) classification, those genes were mainly involved in immune response to virus infection, such as ‘adaptive immune response’, ‘activation of immune response’ and ‘regulation of defense response’ (Supplemental figure 4B). Importantly, most of HSV-1-downregulated genes were enriched in neuronal functions (e.g. memory, cognition, and learning), as well as conduction, transmission and integration of neural signals, furthering strengthening the contribution of HSV-1 in neurodegeneration (Supplemental figure 4C).^1,2^ Besides, KEGG and Reactome pathway analysis of differential expressed genes (DEGs) between the Ctrl and HSV-1 group clearly demonstrated that these DEGs participated in the immune response processes, such as ‘Herpes simplex infection’ and ‘adaptive immune system’ (Figure 3(c) and Supplemental figure 4D). When compared the HSV-1 infection group with the HSV-1+ ABX group, there were only 650 upregulated genes and 361 downregulated genes by ABX (Supplemental figure 4E). GO enrichment analysis indicated that ABX further upregulated these DEGs involved in ‘response to virus’, ‘regulation of defense response’ and ‘cytokine-mediated signaling pathway’, whereas downregulated genes enriched in neuronal functions (Supplemental figure 4 F and 4 G); this supported the critical role of gut microbiota in neurodegeneration.^25^ KEGG pathway also suggested the participation of these DEGs in immune response to virus, such as ‘Herpes simplex infection’, ‘Cytokine-cytokine receptor interaction’, ‘TNF signaling pathway’ and ‘NOD-like receptor signaling pathway’ (Figure 3(d)). Furthermore, the mRNA expression of inflammatory cytokines that were upregulated by HSV-1 infection were significantly enhanced by ABX-mediated depletion of gut microbe (Figure 3(e)). Genes required for the activation of innate immunity, such as Toll-like receptors (TLRs), were also upregulated by ABX (Supplemental figure 4 H). On the contrary, genes related to epigenetic modifications were found to be deregulated in ABX treated group (Supplemental figure 4I). Collectively, the mRNA profiles clearly indicated that gut microbe mainly modulates antiviral immune response to enhance HSV-1-induced inflammation in HSE.

**Figure 4.**
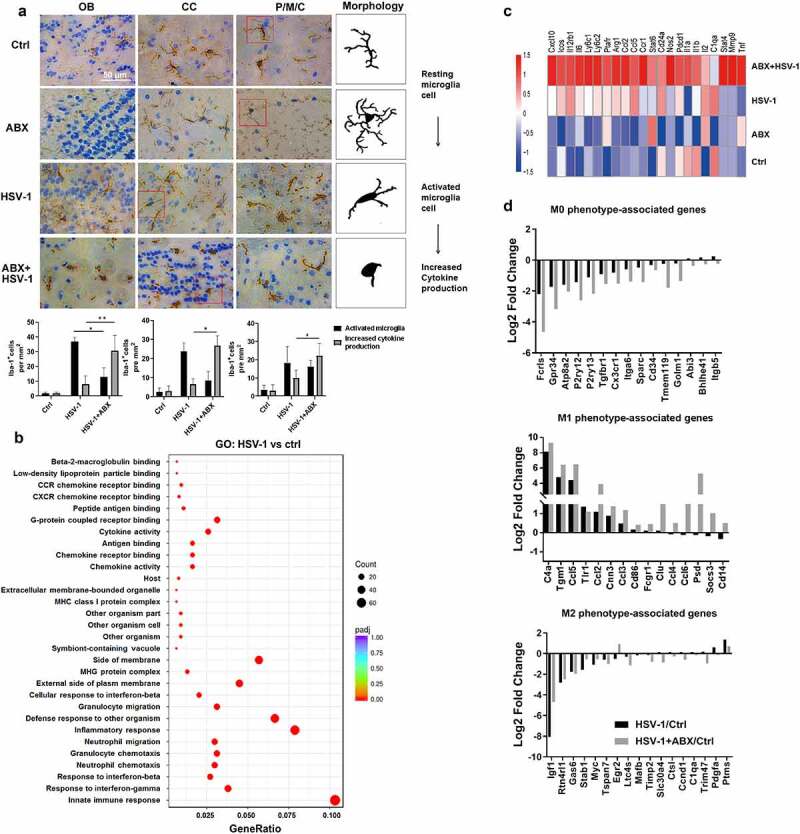
**Lack of microbes enhances microglia immune response**. (a) Representative IHC images of OB, CC, and P/M/C tissue from Ctrl, ABX, HSV-1-infected normal or ABX-treated mice, respectively. Increased Iba-1 staining indicated that HSV-1 infection triggers the transformation from resting microglia to activated microglia, whereas ABX pretreatment induces the microglia to a form with increased cytokine production. Number of Iba-1^+^ ramified parenchymal microglia (active microglia) and microglia in “increased cytokine production” form in different localizations of the CNS were calculated. At least three sections per mouse were examined. Data are presented as mean ± SD. *p< .05 or **p < .01 versus HSV-1 group. (b) Total RNA of sorted mouse CD11b^+^ CD45^lo^ microglia was extracted and the mRNA expression profile was analyzed by RNA-seq. The differentially expressed genes (at least 2-fold, p < .05 in HSV-1 compared with ctrl microglia) were enriched in several key cellular functions, components and biological progresses by Gene Ontology (GO) analysis. (c) Heat map of the expression of microglia activation-related gene transcripts (1.5-fold, p < .05, unpaired *t* test) in sorted microglia from normal, ABX, HSV-1 and ABX + HSV-1 mice. Color code presents linear values. (d) mRNA expression values (log2 fold change vs Ctrl group) of genes from microglia in Ctrl, HSV-1 or HSV-1+ ABX mice were categorized according to the M0, M1 or M2 phenotypes, as described previously.^27^

Notably, several central transcriptional and survival factors of microglia were markedly upregulated by HSV-1, which were further enhanced by ABX (Supplemental figure 4 J). However, characteristic genes related to other cell types, such as neurons, oligodendrocytes, and schwann cells, were significantly downregulated, implying the critical role of microglia in the enhanced susceptibility to HSV-1 infection. Considering that microglia control and coordinate immune reaction to HSV-1 infection in the central nervous system, and the maturation and function of microglia has been reported to be modulated by host intestinal flora,^26^ we then speculated that the absence of intestinal microbes drives microglia susceptible to HSV-1 infection. Indeed, immunofluorescence visualization of Iba-1-labeled microglia showed that the cell morphology of microglia from ABX-treated mice was significantly changed, mainly featuring in dendrite length and cell volume. HSV-1 infection triggered the transformation from resting microglia to activated microglia, whereas ABX treatment induced the microglia to a form with increased cytokine production (Figure 4(a)). Statistical analysis also indicated that the number of microglia in ‘activated microglia’ form was further reduced whereas the “increased cytokine production” form of microglia was increased by ABX treatment. Besides, ABX alone increased the dendrite length, number of branching points and segments of microglia, which was consistent with previous work.^26^ In addition, there were increased prevalence of MHC II-positive microglia, a marker of activated microglia, in ABX-treated mice (Supplemental figure 5A).

To confirm the possible role of microglia activation in ABX-enhanced HSE, we isolated CD11b^+^ CD45^lo^ microglia from Ctrl, HSV-1 and HSV-1+ ABX group mice (Supplemental figure 5B). RNA-sequencing results demonstrated that 443 genes were differential expressed (Supplemental figure 5C), which were enriched in immune response and inflammation response via GO analysis (Figure 4(b)). KEGG analysis also indicated the involvement of ‘Herpes simplex infection’ of these DEGs (Supplemental figure 5D). In addition, a greater number of genes was either upregulated or downregulated by ABX treatment compared to HSV-1 infection, with similar enrichment in KEGG pathways (Supplemental figure 5E and 5 F). Deciphering these DEGs revealed that HSV-1 infection induced the expression of microglia activation-related gene transcripts, which were further enhanced by ABX, implying the hyper-activation of microglia in HSE (Figure 4(c)). Microglia can achieve polarized states that are characterized by specific genetic signatures that allow the distinction of homeostatic (M0), pro-inflammatory (M1), and anti-inflammatory (M2) microglia, respectively.^27,28^ Under HSV-1 infection and ABX treatment, we found that M1-related genes were largely upregulated by HSV-1 infection, whereas M0- and M2-related genes were significantly downregulated, which were further enhanced or reduced by ABX treatment, respectively (Figure 4(d)). These results clearly indicate the M1 polarization of microglia by HSV-1 infection and ABX treatment, consistently with the aggravated inflammation in HSE. Furthermore, HSV-1 infection upregulated several M1-related bacteria and downregulated M2-related bacteria, respectively (Supplemental figure 5 G). Collectively, the absence of intestinal microbes promoted the innate immune response of microglia to HSV-1 infection and accelerated HSE via neuroinflammation.

### 4. Gut microbial metabolite Nicotinamide N-oxide suppresses HSE

**Figure 5.**
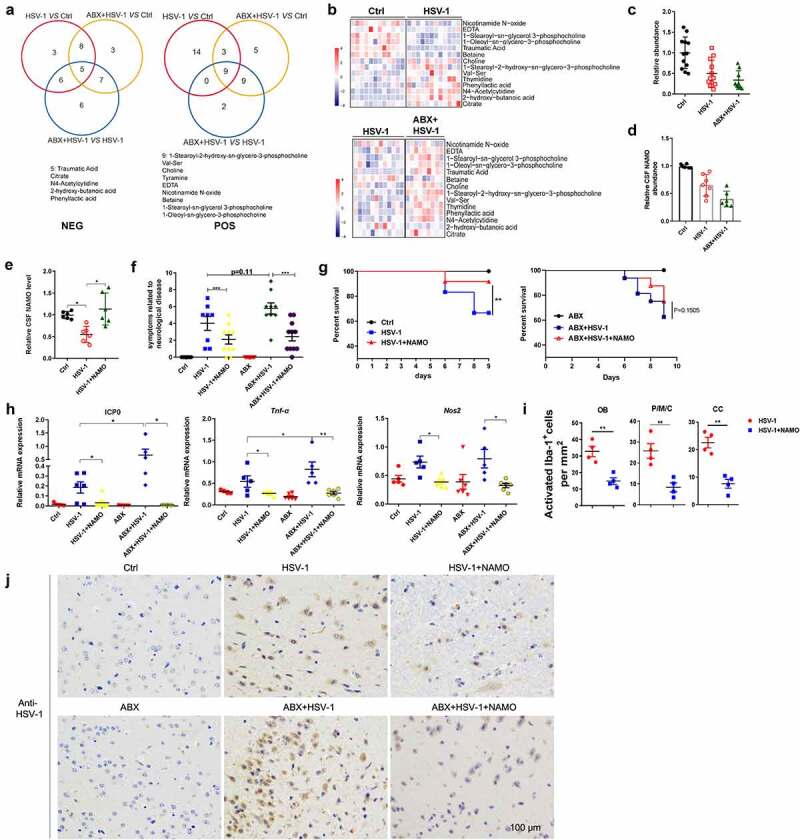
**Gut metabolite Nicotinamide N-oxide suppresses HSE**. (a) Venn diagram showed overlap of significantly differentially regulated metabolites among Ctrl, HSV-1, and HSV-1+ ABX mice in POS model or NES model, respectively. (b) Levels of mutual metabolites involved in all three group were shown. Levels of differential metabolites among Ctrl vs HSV-1, or HSV-1 vs HSV-1+ ABX groups. Color code shows linear values. (c-d) Serum abundance (c) or Cerebrospinal fluid (CSF) levels (d) of NAMO in Ctrl, HSV-1, and HSV-1+ ABX mice. Each symbol represents data from one mice. Data are mean ± SD.       (e) NAMO levels in the CSF of Ctrl, vehicle-treated and NAMO-treated HSE mice. n = 6 mice. *p < .05    , unpaired *t* test. Data are mean ± SD. (f-g) NAMO-treated mice exhibited ameliorative HSE symptoms related to neurological disease score (f) and prolonged survival rate compared with normal or ABX-treated mice (g). n = 6–10 mice per group. **p< .01 or ***p < .001. (h) The mRNA expression of gene *ICP0, TNF-α*, and *NOS2* in OB from different mice was analyzed by qRT-PCR. Data are mean ± SD and are representative of two independent experiments. *p < .05, **p < .01, ***p < .001, unpaired *t* test. (i) Number of activated Iba-1^+^ microglia in different localizations of the CNS from NAMO-treated or vehicle-treated HSE mice. Depicted symbols represent individual mice. At least three sections per mouse were examined. Data are mean ± SD. **p < .01 versus HSV-1 group. (j) Representative IHC images showed the inhibitory effects of NAMO on HSV-1 viral production in OB from NAMO-treated or vehicle-treated HSE mice pretreated with or without ABX. Scale bar, 100 μm.

Next, we interrogated whether gut microbe affected the susceptibility of microglia to HSE by modulating microbiome-associated metabolites. Potential microbiome-associated molecules of serum in WT and HSV-1 infected mice that were previously exposed to PBS or ABX were identified through untargeted metabolomics profiling. PCA analysis of fecal samples from WT, HSV-1 infected and ABX pretreated mice indicated a good quality in both positive (POS) and negative (NEG) ion mode mass spectrometric (Supplemental figure 6A). A total of 6923 metabolites matched to database were identified, and metabolites that had log2 fold change (FC)>1.5 with P < .05 were shown in volcano plots in both POS and NEG mode (Supplemental figure 6B). These altered metabolites were further annotated with the Human Metabolome Database (HMDB) and the KEGG, yielding a total of 38 metabolites (POS model) or 42 metabolites (NES model) that were differential regulated among Ctrl, HSV-1, and HSV-1+ ABX treated mice (Figure 5(a) and Supplemental figure 6C). The 14 mutual metabolites involved in all three group were shown in Figure 5(b). KEGG Pathway enrichment analysis of these metabolites revealed significant changes in amino acid-related metabolic pathways and neurodegeneration-related pathways (Supplemental figure 6D). Metabolite set enrichment analysis (MSEA) also indicated apparent changes in amino acid and lipid metabolic pathways (Supplemental figure 6E). Among these differential regulated metabolites, we found that several short chain fat acids (SCFAs) have been largely increased by HSV-1 infection (Supplemental figure 6 F), in accordance with the increment of SCFA-producing genera (Supplemental figure 2E). However, ABX treatment did not affect their production.

We then investigated the possible connection between amino acid metabolism and HSE, and found the significant alternation of metabolites in glycine, tryptophan, phenylalanine, tyrosine, and nicotinamide (NAM) metabolism (Supplemental figure 7A and 7B). Interestingly, several key intermediates or final products of these metabolic pathways, such as tryptophan, tyrosine, dopamine and nicotinamide N-oxide (NAMO), were largely reduced by HSV-1 infection. On the contrary, the abundance of several by-products, such as tyramide, phenyllactic acid, and indolelactate, were significantly upregulated. The ABX treatment further enhanced the abundance of these by-products and reduced the levels of final products, respectively (Supplemental figure 7B). Apparently, there was a marked switch from tryptophan-NAM transformation to tryptophan-phenylalanine-tyramide transformation in ABX treated mice when compared with normal HSV-1-infected mice, resulting in the reduced NAMO and increased phenylalanine abundance (Supplemental figure 7B). Through the kynurenine pathway, tryptophan can be metabolized into nicotinamide adenine dinucleotide (NAD^+^), a critical regulator maintaining physiologic processes (Supplemental figure 7C). NAD^+^ can also be de novo synthesized from nicotinic acid (NA) by the Preiss-Handler pathway, while most NAD^+^ is recycled via salvage pathways from NAM. Excess NAM is mainly metabolized by nicotinamide N-methyltransferase and subsequently aldehyde oxidase to pyridone carboxamides. NAM is also oxidizes by unknown enzyme to NAMO. Our results showed that the abundance of NAMO was largely reduced by HSV-1 infection and ABX treatment, suggesting the possible role of dysregulated NAM metabolism in HSE (Supplemental figure 7B).

To screen for candidate HSE-regulating metabolites, we used microglia BV2 cells *in vitro* to analyze these differential-regulated metabolites that fit the following criteria: 1) deregulated in both HSV-1 and HSV-1+ ABX group; 2) abundance change in HSV-1 infected mice was further enlarged by ABX treatment; 3) Anti-inflammatory effect of downregulated metabolite and pro-inflammatory activity of upregulated metabolite. We firstly examined the time kinetic of inflammatory cytokine induced by HSV-1 infection or LPS treatment (Supplemental figure 8A and 8B). We then analyzed the regulatory functions of different metabolites by detecting the levels of cytokine *TNF-α* and *NOS2* at 6 or 9 h. Notably, among these metabolites, we focused on NAMO for the following reasons: one of the final product of NAM metabolism originated from tryptophan metabolism (Supplemental figure 7A-C); one of the 14 mutual metabolites (Figure 5(b)); the abundance largely reduced by HSV-1 and further reduced by ABX treatment (Figure 5(c)); potent anti-inflammatory activity against HSV-1-induced inflammation in vitro cell model (Supplemental figure 8C). Finally, we analyzed the *in vivo* levels of NAMO via mass spectrometry and found that NAMO in the cerebrospinal fluid (CSF) was significantly lower in HSV-1 infected and much lower in ABX-treated HSV-1 infected mice (Figure 5(d)).

To causally link a decrease in NAMO levels with the associated HSE phenotype, we continuously supplemented HSV-1-infected mice in the presence or absence of ABX-treatment with NAMO. We found that NAMO was able to cross the blood-brain barrier (Supplemental figure 8D), and the NAMO levels were significantly increased in the CSF compared with water-treated controls (Figure 5(e)). Notably, NAMO-treated mice exhibited ameliorative HSE symptoms related to neurological disease score (figure 5(f)) and prolonged survival rate (Figure 5(g)). NAMO treatment also reduced the expression of viral genes and inflammatory cytokines in OB (Figure 5(h)). In addition, the number of activated microglia in OB, P/M/C and cortex was significantly reduced by NAMO (Figure 5(i)). Histopathological analysis also demonstrated that NAMO markedly reduced HSV-1 virus production (Figure 5(j)). Together, these results demonstrated that gut microbiota-derived NAMO suppresses neuronal inflammation to prevent HSE.

### 5. Nicotinamide N-oxide inhibits microglia activation via activating mitophagy

**Figure 6.**
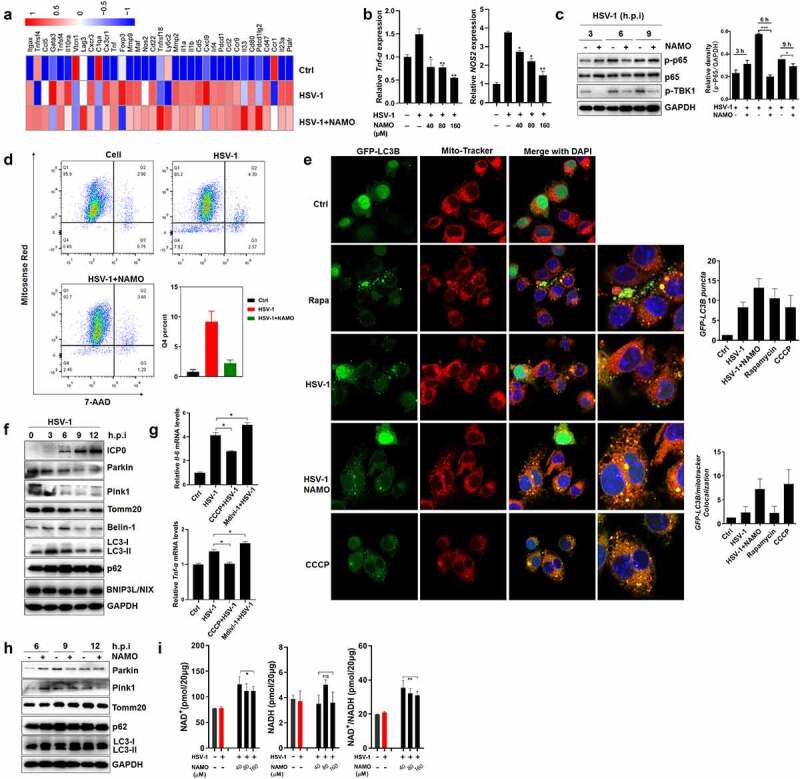
**Nicotinamide N-oxide inhibits microglia activation**. (a) BV2 cells were infected with HSV-1 (MOI = 1) in the presence or absence of NAMO (40 μM) for 12 h and total RNA was subjected to RNA sequencing. Differentially expressed inflammatory genes (at least 1.5-fold, p < .05) were shown in heat map. Color code presents linear scale. (b) qRT-PCR of gene TNF-α and NOS2 in BV2 cells 9 h after HSV-1 and NAMO treatment. Data are mean ± SD from at least three independent experiments. Significant differences set as *p < .05, **p < .01, ***p < .001. (c) BV2 cells were infected with HSV-1 (MOI = 1) in the presence or absence of NAMO (40 μM) at indicated time and total protein was subjected to western blot assay. The band intensity of p-p65/GAPDH was quantified by Image J software. (d) BV2 cells stained with Mitosense Red7/7-AAD were analyzed by flow cytometry. Representative cytometry graphs were shown and the percentage (%) of negatively labeled Mitosense Red7/7-AAD were depicted. Data are mean ± SD from three independent experiments. (e) BV2 cells were transfected with plasmid GFP-LC3 (2 μg) for 24 h before HSV-1 (MOI = 1) infection and treatment with Rapamycin (10 μM), NAMO (40 μM) or CCCP (10 μM) for 12 h. The cells were then fixed, stained with Mito-Tracker (red) and DAPI (blue) for 1 h. Fluorescent images were captured by a confocal microscopy. The number of yellow (GFP/mito-tracker colocalization) or GFP-LC3B puncta was also quantified. At least 50 cells from 5 representative fields were counted in each independent experiment. Scale bar: 10 μm. (f) BV2 cells were infected with HSV-1 for indicated times and targeted proteins were analyzed by western blot. Effects of on autophagy-related proteins. (g) qRT-PCR of *IL-1β* and *TNF-α* in BV2 cells 9 h after HSV-1 exposure in the presence of Midiv-1 (20 μM) or CCCP (10 μM). Data are mean ± SD. (h) Effects of NAMO (40 μM) on mitophagy-related proteins. (i) BV2 cells were infected with HSV-1 in the presence of NAMO for 12 h, and relative NAD+, NADH and NAD+/NADH levels were determined. Data are mean ± SD (n = 3 independent experiments), with the significance as *p < .05, **p < .01, ***p < .001 versus HSV-1 group.

To explore potential downstream mechanisms by which NAMO ameliorate HSE progress, the preventive effect of NAMO on microglia-mediated neuro-inflammation was tested. We firstly examined whether NAMO directly inhibited HSV-1 infection in microglia BV2 cells to prevent the HSV-1-induced inflammation. We analyzed the mRNA expression profiles of BV2 cells infected with HSV-1 for 24 h in the presence or absence of NAMO by RNA-seq. Indeed, HSV-1 infection caused the enrichment of DEGs in cytokine production, inflammation and immune response (Supplemental figure 9A and 9B). There were 53 genes significantly upregulated and 120 genes downregulated (P < .01 and twofold change) upon NAMO treatment in HSV-1 infected BV2 cells (Supplemental figure 9C). Among these DEGs, we identified that NAMO triggered opposite expression patterns of several central microglia transcripts and survival factors, as well as genes linking to microglia M0/M1/M2 phenotypes (Supplemental figure 9D). Besides, microglia activation and cytokine release were apparently diminished by NAMO treatment (Figure 6(a,b)). In addition, NAMO inhibited the activation of the DNA-sensing cGAS-STING pathway, the mainly anti-HSV-1 immune signaling in microglia^11^ (Supplemental figure 9E). NAMO treatment also inhibited the phosphorylation of TBK1 and p65, two key regulators in antiviral immune recognition and inflammation (Figure 6(c) and Supplemental figure 9 F). Finally, we isolated primary microglia and found that similarly as BV2 cells, NAMO treatment only slightly inhibited HSV-1 infection, whereas dramatically reduced neuronal toxic cytokine production (Supplemental figure 9 G). Together, these above results suggested that NAMO inhibited microglia maturation and activation to ameliorate HSE.

The activation of cGAS-STING signaling requires functional mitochondria and mitochondrial dysfunction may cause inflammation and microglia activation.^29,30^ By Mitosense Red7/7-AAD staining and FACS assay, we found that HSV-1 infection caused damaged mitochondria (Mitosense Red7 negative and 7-AAD negative) and slightly triggered cell death (7-AAD positive) (Figure 6(d) and Supplemental figure 10A). We then examined the effect of HSV-1 infection on mitophagy, the mainly cellular process for the clearance of damaged mitochondria. Indeed, HSV-1 triggered the formation of GFP-LC3 puncta, a marker of enhanced autophagosomes (Figure 6(e)). However, only limited GFP-LC3 puncta co-localized with mitochondria, similar to the treatment of autophagy inducer rapamycin. On the contrary, mitophagy inducer CCCP triggered the co-localization between GFP-LC3 puncta and damaged mitochondria (Figure 6(e)). In addition, the protein levels of autophagy marker LC3-II and autophagic substrate p62 were largely upregulated, whereas mitophagy-related proteins, including Parkin, Pink1, and Tomm20, were significantly downregulated (figure 6(f)). Considering that Parkin and Pink1 are mediators interacting with several substrates to initiate and promote mitophagy, and their deficiency always result in mitochondrial dysfunction, these results suggested that HSV-1 infection inhibits mitophagy. To clearly clarify whether HSV-1 downregulates Pink1/Parkin to inhibit mitophagy, or activates mitophagy to promote the degradation of Pink1/Parkin, we examined the effects of mitophagy inducer CCCP and inhibitor Mdivi-1, respectively. As shown in supplemental figure 10B and 10C, CCCP treatment significantly upregulated the mRNA and protein levels of Pink1/Parkin, which were inhibited by HSV-1 infection. Besides, CCCP lose the ability to induce mitophagy in HSV-1-infected cells as evidenced by the reduced LC3-II levels, indicating that HSV-1-mediated inhibition on the de novo synthesis of Pink1/Parkin apparently prevents the Pink1/Parkin-dependent mitophagy by CCCP. In addition, mitophagy inhibitor Mdivi-1 failed to restore the protein levels of Pink1/Parkin (Supplemental figure 10D), ruling out the possibility that HSV-1 reduces Pink1/Parkin levels via activating mitophagy. To further confirm the inhibitory effect of HSV-1 on mitophagy, we expressed mitochondrial matrix–localizing Keima (mito-Keima) in BV2 cells. Mito-Keima is a pH-sensitive fluorescent protein that exhibits green color within the mitochondrial matrix whereas exhibits red color within the acidic lysosome after mitophagy. As shown in Supplemental figure 10E, CCCP treatment resulted in a certain number of acidic puncta whereas only little ignorable red dots appeared upon HSV-1 infection. CCCP treatment also inhibited the activation of microglia induced by HSV-1 infection (Figure 6(g)). On the contrary, mitophagy inhibitors mdivi-1 enhanced the production of inflammatory cytokines (Figure 6(g)). Therefore, these above results clearly indicated that HSV-1 infection caused mitochondria dysfunction and inhibited mitophagy-mediated clearance to activate microglia and inflammatory cytokine release.

Importantly, we observed that NAMO treatment apparently reduced the percent of damaged mitochondria (Figure 6(d)), and stimulated the colocalization between GFP-LC3 and mitochondria (Figure 6(e)), implying the involvement of activated mitophagy in removing damaged mitochondria. In addition, NAMO reduced the protein levels of p62 and upregulated LC3-II (Figure 6(h)). We also isolated mitochondria and found that similarly, NAMO decreased p62 and increased LC3-II levels in mitochondria, the phenomenon of which was eliminated by lysosomal inhibitor bafilomycin A1 (Supplemental figure 10 F). Furthermore, NAMO significantly upregulated the red puncta of mito-Keima (Supplemental figure 10E). Therefore, the above results clearly indicated that NAMO activates a completely and functional mitophagic flux. However, NAMO did not enhance the expression of Parkin and Pink1 to activate mitophagy (Figure 6(h)). NAD+ maintains mitochondrial homeostasis, and boosting cellular concentration of NAD+ has been well demonstrated to stimulate mitophagy-mediated clearance of damaged mitochondria and consequently inhibit the release of pro-inflammatory cytokines.^31^ We observed that HSV-1-infected cells exhibited a similar NAD+/NADH ratio compared with control cells, while NAMO significantly upregulated the NAD+/NADH ratio and NAD+ levels (Figure 6(i)). Interestingly, both NAMO precursors, NAM and NA, were also able to reduce mitochondrial damage and enhance NAD+/NADH ratio (Supplemental figure 10 H), consistent with previous reports that NA and NAM activate mitophagy via NAD+.^31^ Additionally, we analyzed the possible involvement of autophagic protein ULK1, which has been reported to mediate NAD+-dependent mitophagy.^31^ Knockdown of ULK1 by siRNA restored the expression of p62 and Tomm20 while reduced the levels of LC3-II modulated by NAMO (Supplemental figure 10I and 10 J), further strengthening the necessary role of NAD+ in NAMO induced mitophagy. In sum, NAMO triggered NAD+-dependent mitophagy to remove damaged mitochondria and thereby inhibit microglia activation and inflammation.

Furthermore, considering that HSV-1 infection causes neuron damage via directly triggering neuronal cell death or indirectly through environmental neuronal toxic factors released by other cells, such as microglia, we also examined the protective effect of NAMO on HSV-1-infected neuron cells N2A. We found that NAMO inhibited virus production, viral gene and protein synthesis (Supplemental figure 11A-11C), as well as restored cell morphological change caused by HSV-1 (Supplemental figure 11D). By time-of-addition assay, we found that NAMO mainly inhibited HSV-1 early infection (Supplemental figure 11E). NAMO affected viral adsorption, while had no effect on viral penetration (Supplemental figure 11 F). Detection of cell membrane-associated viral glycoprotein gB by FACS also indicated a reduced viral adsorption (Supplemental figure 11 G). Accordingly, pretreatment with NAMO reduced HSV-1 infection (Supplemental figure 11 H). Furthermore, conditional medium of HSV-1-infected BV2 cells triggered cell death of N2A cells, which were still protected by NAMO treatment (Supplemental figure 11I). Finally, treatment with NAMO precursors, such as NAM and NA, also reduced the production of inflammatory cytokines and inhibited HSV-1 gene expression in neuron cells (Supplemental figure 11 J and 11 K). Taken together, these above results clearly indicated that NAMO suppressed HSE through at least two mechanisms acting in a paracrine manner: activating mitophagy to suppress microglia activation, and preventing neuron damage via inhibiting HSV-1 early infection and reducing microglia-mediated neuronal cytotoxic microenvironment.

### 6. Nicotinamide N-oxide is generated by neomycin-sensitive bacteria

**Figure 7.**
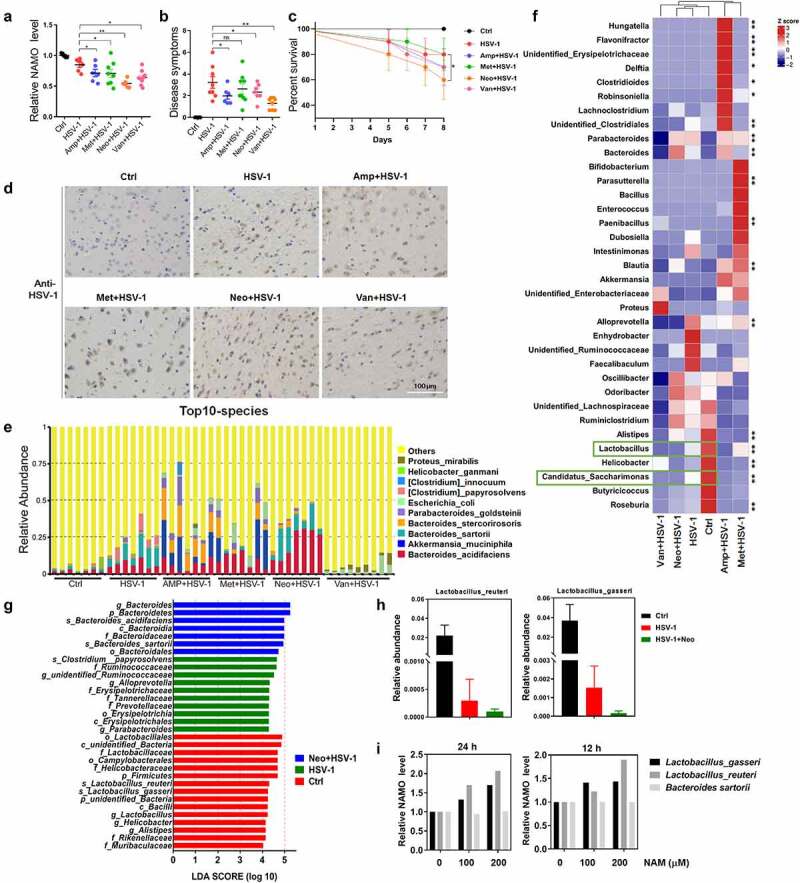
**Nicotinamide N-oxide is generated by neomycin-sensitive bacteria**. (a) Serum NAMO levels of HSV-1-infected mice pretreated with vancomycin (van), ampicillin (amp), neomycin (neo) or metronidazole (met), respectively. Each symbol represents data from one mouse. n = 6–10 mice per group. Data are mean ± SD. *p < .05, **p < .01, or ***p < .001 versus HSV-1 group by *t* test. (b-d) Neomycin-pretreated mice exhibited aggregated HSE symptoms related to neurological disease score (b), shortened survival rate compared with normal or ABX-treated mice (c), and enhanced virus production in OB as revealed by IHC assay (d). n = 6–10 mice per group. *p < .05, **p < .01, or ***p < .001 versus HSV-1 group by *t* test. Scale bar, 100 μm. (e) Top 10 microbes at the species level in fecal samples from Ctrl, HSV-1 and HSV-1 + antibiotic mice, respectively. n = 6–10 mice per group. (f) Metastats analysis of the correlation between the altered bacteria in genus level. Colors on the heatmap indicate the relative abundance. Red indicates bacteria that are upregulated, and blue represents downregulated. Green squares indicate bacterial genus whose levels were significantly downregulated by HSV-1 but further reduced by neomycin. **p < .01, ***p < .001 versus HSV-1 group. (g) LEfSe analysis showed the taxa most differentially abundant among ctrl, HSV-1 and HSV-1+ Neo groups. Only taxa meeting an LDA significant threshold value of ≥ 2.0 are shown. (h) Relative abundance of bacterial species *Lactobacillus_gasseri* and *Lactobacillus_reuteri* in feces samples from Ctrl, HSV-1 and HSV-1+ Neo group. (i) NAMO levels in bacterial supernatant. Bacterial cultures of *L. gasseri, L. reuteri* and *B. sartorii* were incubated with NAM for 12 h or 24 h at 37°C. NAMO was then detected by UPLC–MS/MS. Data are mean ± SD (*n* = 5). Significance as *p < .05, **p < .01, ***p < .001 by *t* test.

Finally, to clarify the origin of NAMO produced by gut microbiota, single antibiotic vancomycin, ampicillin, neomycin, or metronidazole was adopted to pretreat mice before HSV-1 infection, respectively. We found that single antibiotic treatment reduced the NAMO level in CSF (Figure 7(a)), exhibited similar HSE phenotypic scores except for vancomycin (Figure 7(b)), enhanced the mortality of HSV-1-infected mice (Figure 7(c)) and enhanced HSV-1 virus production (Figure 7(d)). Among these antibiotics, neomycin exhibited the biggest weight loss (supplemental figure 12A), the highest neuronal inflammation in OB, P/M/C, and CX (supplemental figure 12B), the highest NAMO reduction and HSE severity (Figure 7(a,b)), suggesting that NAMO was mainly generated by neomycin-sensitive bacteria.

Next, we analyzed the changes produced by single antibiotic treatment in the gut microbiota. Pairwise comparisons using the permutational multivariate analysis of variance (PERMANOVA) test and Anosim analysis indicated a statistically significant separation between groups (p < .005). Indeed, neomycin treatment exhibited the most similarity with HSV-1 infected mice in alpha or beta diversity, and in PCoA analysis (Supplemental figure 13A-C). The top 10 microbes at the phylum and genus levels also indicated the similar variations in the composition of the gut microbiota between HSV-1 and HSV-1+ Neo group (Supplemental figure 13D and 13E). Analyses of the microbiota at the phylum level revealed a dominance of *Bacterioidetes* and *Firmicutes* in all the groups. Moreover, the abundance of the top 12 bacterial species was largely reduced by HSV-1 infection and antibiotics treatment, except for *B. sartorii* and *B. acidifaciens*, which were conversely increased by HSV-1 infection and were further enhanced by neomycin treatment (Figure 7(e) and Supplemental figure 13 F), consisting with previous result (Supplemental figure 2 F).

In addition, PICRUSt functional enrichment analysis of these differential regulated microbiota suggested the similar trend of HSV-1 and HSV-1+ Neo group mainly in amino acid and energy metabolism (Supplemental figure 14A and 14B). Neomycin treatment reduced nucleotide metabolism, replication and repair process, whereas enhanced carbohydrate and amino acid metabolism. By focusing on amino acid metabolism, we found that HSV-1 infection reduced metabolisms of most amino acids while upregulated phenylalanine metabolism (Supplemental figure 14C and 14D). On the contrast, neomycin further upregulated phenylalanine metabolism and recovered the reduction of other amino acid metabolisms, except for nicotinate and nicotinamide metabolism that was further reduced, suggesting a shift from tryptophan-NAM to tryptophan-phenylalanine transformation (Supplemental figure 14D). Therefore, these PICRUSt prediction results suggested that neomycin-sensitive bacteria regulated NAM and NAMO metabolism to counteract with HSE.

Next, we sought to identify the specific bacterial species in the HSV-1 group whose abundance was altered by neomycin. We used metastats analysis to identify the correlation between these phenotypes and the altered bacteria in genus level (figure 7(f)). When compared with Ctrl group, only *Lactobacillus* and *Candidatus_Saccharimonas* were significantly negatively correlated with HSV-1, which were further reduced by neomycin treatment (Supplemental figure 13 G), suggesting their possible roles in NAMO production. Among these reduced bacteria, only the ratios of *Lactobacillus_gasseri* and *Lactobacillus_reuteri* were apparently deregulated by neomycin treatment when compared with HSV-1-infected mice, which were consistent with the results of LEfSe (LDA Effect Size) analysis (Figure 7(g,h)). Accordingly, we finally examined whether *L. gasseri* and *L. reuteri* were able to catalyze the transformation of NAMO from NAM *in vitro* (Supplemental figure 7C). We also tested the possible NAMO-production function of *B. sartorii*, one of the top10 species (Figure 7(e)). NAM was added to the culture medium of *L. gasseri, L. reuteri, and B. sartorii*, respectively, and the released NAMO was detected by MS. As shown in Figure 7(i), both *L. gasseri* and *L. reuteri* were able to generate NAMO via NAM, whereas *B. sartorii* was unable to transform NAM. Besides, *L. reuteri* had a higher catalytic activity toward NAM than *L. gasseri*. Further works are inspired to examine whether NAMO was transformed from NAM by both *L. gasseri* and *L. reuteri in vivo*. In sum, above results indicated that NAMO was mainly generated by neomycin-sensitive bacteria, especially *L. gasseri* and *L. reuteri*.

## Discussion

We have established a previously unknown role for the intestinal microbiome in restricting immune response to neurotropic virus HSV-1 infection and HSE progression. Our works reveal that: (1) HSV-1 neurotropic infection causes gut microbiota dysbiosis, which is partly reversed by ACV treatment; (2) loss of the gut microbiota leads to aggregated HSV-1 burden in the CNS and HSE pathology, which is associated with hyper-activated microglia-mediated neuroinflammation; and (3) NAMO, a microbial metabolite mainly produced by neomycin-sensitive bacteria, especially *L. gasseri* and *L. reuteri*, can active mitophagy in microglia to regulate microglia homeostasis and prevent HSV-1 early infection in neuronal cells, which in turn suppress HSE progression.

Although an altered composition of intestinal microbiota had long been recognized in an individual with numerous virus infections,^14,32–34^ microbiota modulates the host antiviral immune response to virus infection through as-yet unclear mechanisms. In particular, whether gut microbiota is involved in the CNS response to neurotropic virus infection remains uncertain. Herein, our study indicates that brain infection of neurotropic herpesvirus HSV-1 leads to gut microbial dysbiosis. In detail, HSE mice showed a great ratio of *Cyanobacteria* at the phylum level and high abundance of the genus *Bacteroides, Odoribacter, Rikenellaceae*_*RC9_gut_group*, and *Ruminococcaceae_UCG-014* (Figure 1(d,e)). Besides, these differential microbes could be largely restored by the anti-HSV-1 drug ACV, suggesting their closely associations with the development of HSE. The altered microbiota exhibited virus-specific tropism that were different from other viruses, such as SARS-CoV-2 and IAV.^32–34^ For instance, the predominant respiratory microbial taxa of severely COVID-19 patients were *Burkholderia cepacia complex*, S*taphylococcus epidermidis*, and *Mycoplasma spp*.^33^ On the other hand, although the mechanism linking brain infection of HSV-1 and gut microbial dysbiosis has not been clarified, one possible explanation is HSV-1 neuronal infection affects the components and functions of gut microbes through the gut-brain axis and the neural regulation pathway, in which the vagus nerve links between the gut and the spinal cord (autonomic nervous system).^35^ The ended vagus nerve of brain stem nuclei receiving and giving afferent and efferent fibers may regulate the gut functions.^35^ In addition, the viral spread of HSV-1 to enteric neurons from brain also cause gastrointestinal neuromuscular dysfunction and gut dysmotility, which further affect gut microbes.^36,37^ Consistently, we examined the intestinal inflammation upon HSV-1 intranasal infection and found that HSV-1 significantly upregulated the production of inflammatory cytokines in gut (Figure S1F). Increasing evidence clearly indicates that the intestinal inflammation and gut microbiota are reciprocally regulated.^22,23^ Therefore it is reasonable that HSV-1-induced dysbiosis alters the composition of microbial metabolites (Figure 5(b)), increases the production of enterotoxins and intestine permeability, compromises immune response and energy metabolism, resulting in intestinal inflammation. On the contrary, the enhanced intestinal inflammation by HSV-1 neurotropic infection might remodify the ratio of harmful/beneficial bacteria (figure 1(f) and supplemental figure 2E), provide optimal genetic or environmental conditions for the growth of pathological bacteria, promoting their bidirectional regulation of immune responses in the gut and brain via the Gut-Brain-axis. Together, our study showed a unique character of microbiota alteration contributed by neurotropic virus infection.

It is well recognized that microglia play critical roles in the development of HSE,^5,11,12^ and the detailed regulatory mechanism remains unclear. Consistently, we showed here that upon HSV-1 early infection, microglia is activated, with changed immune properties and cell numbers, to stimulate antiviral immune response (Figures 3,4). Besides, depletion of gut microbiota by ABX treatment primed activated microglia to proinflammatory state, resulting in enhanced susceptibility to HSV-1 brain infection and HSE progression, similar to the prior findings of ABX-treated mice challenged with IAV and HBV.^15,16,21^ Actually, the microglial immune response plays dual roles in HSE.^5^ For instance, microglia stimulate cGAG-STING-dependent production of type I IFN and enable antiviral state of other cell types to restrict HSV-1 early infection.^11^ However, excessive activation of microglial immune response leads to pathology due to the closely crosstalk between inflammatory response and innate antiviral response at the convergence of the NF-κB and IRF signaling pathways.^5,38,39^ Therefore, the negative regulation of immune response is crucial for maintaining immune homeostasis to ameliorate HSE symptoms. Notably, gut microbiota has been demonstrated to modulate the maturation and activation of microglia,^26,40,41^ and by RNA-seq analysis, we found that the deficiency of gut microbiome causes the hyperactivation of microglia, which contribute substantially to the exaggerated neuroinflammation and HSE-associated death, implying gut microbiota as negative brake of microglia activation. Similarly, another previous work also demonstrated that gut microbiota restricts microglia-mediated pro-inflammatory and neurotoxic activities via the aryl Hydrocarbon Receptor signaling.^42^

To explore the mechanistic relationship linking gut microbiota to microglia activation and restricted neuroinflammation, we focused on altered microbial metabolites in response to HSV-1 infection. By non-targeted metabolomics technique, we revealed that these dysregulated metabolites are mainly involved in amino acid metabolism, particularly phenylalanine-tryptophan-related pathways (Supplemental figure 7A and 7B). HSV-1 infection selectively switched tryptophan-NAM transformation to tryptophan-phenylalanine transformation, leading to significantly increased abundance of phenylalanine. Notably, abnormal production of phenylalanine by gut microbiota was tightly associated with AD progression,^43^ and CNS infection of HSV-1 has been extensively implicated in the onset and progression of neurodegeneration, typical of AD.^1,44^ It is therefore reasonable that the dysregulated amino acid metabolism by HSV-1 is a key causative factor contributing to AD and other neurodegenerative diseases. Our RNA-seq results also confirmed the potent influence of HSV-1 on neurological disorders (Supplemental figure 4B and 4C). In addition, we found that most microbiota-derived metabolites inhibit IFN-based innate antiviral response in microglia, implying that the host usually orchestrates numerous negative feedback loops of innate antiviral responses to avoid immune pathologies during virus late infection.^45^ Among these differential regulated metabolites, we identified NAMO, derived from neomycin-sensitive bacteria, acting as a crucial mediator limiting the inflammatory response of microglia to balance CNS immune response initiated by HSV-1 infection. Exogenous administration of NAMO to ABX-treated HSE mice diminished microglia-mediated proinflammatory responses and restricted HSV-1 infection in CNS (Figure 5). Interestingly, the protective effect of NAMO on HSE seems weaker in ABX-treated mice than in SPF mice (Figure 5(g)), probably due to the strong impact of ABX on promoting HSE. However, NAMO reduced the levels of pro-inflammatory cytokines and virus production in ABX-treated mice to a similar extent as that of in SPF mice (Figure 5(h,j)). Future works are urgent to test the effect of a complete fecal microbiota replacement or other defined microbial communities in addition to NAMO alone. Importantly, gut microbiota-derived SCFAs have been reported to positively modulate microglia maturation, morphology and function,^26^ whereas we confirmed NAMO as negative regulator restricting microglia activation, implying the cooperation of NAMO and SCFAs to maintain microglia homeostasis and immune status. Furthermore, few studies have highlighted that microbial metabolites, mainly SCFA and bile acids, shape type I IFN response of different immune cell types to regulate virus infection,^46,47^ additional studies are thus needed to evaluate whether other CNS cells, such as regulatory CD4+ and CD8 + T cells^48^ also may be required to mediate the anti-inflammatory response of NAMO on HSE. Of note, most metabolites known to inhibit the inflammatory response were derived from dietary tryptophan, such as I3S and TDD,^42^ whereas NAMO had different source and chemical structure, suggesting the complexity and diversity of commensal microbiota-mediated regulation on host immune response.

Damaged mitochondria are critical mediators of both immune activation and inflammation in neuropathogenesis,^49,50^ and are specifically eliminated by mitophagy, one type of selective autophagy that maintains mitochondrial quality control and homeostasis via the autophagic machinery.^51^ Mitochondria are also required for the successful RIG-I-MDA5-MAVS antiviral signaling pathway, and different viruses adopt various strategies to alter mitochondrial function to circumvent host innate immune responses and cause neuronal damage.^52^ Indeed, mitophagy is gradually recognized as a promising therapeutic target for virus infection-associated diseases, including those patients of COVID-19.^53,54^ In our study, we determined the connection between HSV-1-mediated mitochondrial damage, mitophagy and microglial activation, which was consistent with previous work.^29^ We showed that HSV-1 activates microglia by increasing mitochondrial damage via defective mitophagy, whereas microbial metabolite NAMO restores NAD+-dependent mitophagy to remove damaged mitochondria and thereby inhibits microglia activation and inflammatory response. HSV-1 infection induced time-dependent accumulation of autophagic marker LC3-II and substrate p62, downregulated the expression of mitophagy marker PINK1 and PRKN, and interrupted the colocalization between GFP-LC3 puncta and mitochondria, indicating the blockage of the mitophagic flux. On the contrary, NAMO promoted the formation of mitophagososme to reduce damaged mitochondria and inflammatory cytokine production. Interestingly, we demonstrated the unclarified function of NAMO in NAD+ production and further works are required to determine the precise mechanism of NAMO in NAD+-mediated mitophagy regulation.^31^ Whether CXCR2, a previously confirmed target of NAMO,^55^ participates in such a process is interesting to be examined.

Finally, we revealed that NAMO is mainly generated by neomycin-sensitive bacteria, especially *L. gasseri* and *L. reuteri*, with the following reasons: 1) Both NAMO level and *L. gasseri* and *L. reuteri* abundance are significantly reduced in HSE mice; 2) *L. gasseri* and *L. reuteri* can metabolize NAM into NAMO *in vitro*; 3) The neomycin-treated mice display a reduced abundance of *Lactobacillus* and NAMO, which jointly confer a susceptibility to HSV-1 infection. Actually, several other specific gut microbes, such as *Akkermansia muciniphila*, have been demonstrated to regulate the NAM metabolism.^56,57^ For instance, *A. muciniphila* modulated NAM synthesis to ameliorate neurodegenerative disorders.^56^ We found that the abundance of *A. muciniphila* does not significantly reduced by HSV-1 infection, suggesting the involvement of different regulatory mechanisms of NAM in HSE. In addition, both *L. gasseri* and *L. reuteri* are well recognized as probiotics to immunomodulate the innate and adaptive systems, to shape the maturation and function of immune cells, and to prevent virus infection.^58–61^ However, the specific microbial metabolites or components mediating their protective functions have not been clarified. Future works are required to determine whether supplement with *L. gasseri* and *L. reuteri* can inhibit HSV-1 neurotropic infection and HSE progression *in vivo*. Finally, our study is limited by a lack of GF mice, a model that could exclude the off-target antibiotic^62^ and provide golden evidence supporting the function of NAMO as a microbial metabolite in the regulation of host inflammatory response. The phenotype observed in this study may be not an off-target of antibiotics like aminoglycoside antibiotics,^62^ as we found that neomycin-sensitive microbiota can metabolize NAM into NAMO *in vitro* and neomycin-treat mice are susceptible to HSV-1 infection.

In summary, our study uncovers an unappreciated role of gut microbial metabolite in ameliorating HSE via inhibiting microglia hyper-activation to maintain immune homeostasis and avoid immunopathology. The altered microbiota composition and metabolite configuration by HSV-1 neurotropic infection, as well as the finding that NAMO restricts HSV-1 dissemination in the CNS via activating mitophagy in microglia, may provide insight into the HSE pathogenesis and enable approaches for mitigating HSV-1-related disease and possibly, its casually linked neurodegenerative disorders.

## Materials and methods

### Cell, virus, and regents

BV2 cells and N2A cells were purchased from the Cell Bank of the Chinese Academy of Sciences (Shanghai, China). Vero cell line (ATCC) and BV2 cell were cultured in Dulbecco’s modified Eagle’s medium (DMEM; GIBCO) with 10% fetal bovine serum (FBS; GIBCO). The N2A cells were cultured in Eagle’s minimal essential medium (MEM; GIBCO) supplemented with 10% FBS. HSV-1 F strain (ATCC) was a present from Hong Kong University initially, propagated in Vero cells and preserved in −80°C. Enhanced Green fluorescent protein (EGFP)-tagged HSV-1 F strain (EGFP-HSV-1) (EGFP-tagged viral protein Us11) was obtained from the research group of Professor Yuan Li (Jinan University, China).

Acyclovir (ACV) was purchased from Sigma-Aldrich (St. Louis, MO, USA). Mdivi-1 (HY-15886) was purchased from MedChemExpress (New Jersey, USA). mitoKeima was purchased from mKeima-RedMito-7 plasmid (#56018) were obtained from Addgene (Cambridge, MA, USA). Nicotinamide N-oxide (S4785), nicotinamide (S1899), and nicotinic acid (S1744) were purchased from Selleck Chemicals (Houston, TX, USA). Antibodies, including anti-gB (ab6506), anti-ICP0 (ab6513), and anti-VP5 (ab6508) were purchased from Abcam (Cambridge, UK), anti-β-actin (GTX109639) were purchased from GeneTex, anti-GAPDH (2118), anti-p-p65 (3033), anti-p65 (8242), anti-p-TBK1 (5483), anti-LC3B (3868), anti-p62 (88588), anti-Parkin (4211), anti-Pink1 (6946), anti-Tomm20 (42406), anti-Beclin-1 (3495), and anti-BNIP3L/NIX (44060) were obtained from Cell Signaling Technology (Danvers, MA, USA).

### Mice

All male mice were purchased from Guangdong medical laboratory animal center at 4 weeks of age, and began experiment at 5 weeks of age. All mice were kept on a strict 24-h reverse light-dark cycle, with lights being turned on from 7:00 to 19:00. For HSV-1 infection model, five-weeks-old mice were infected with 2 × 10^6^ PFU HSV-1 by nasal intubation drip. Mock infected mice were treated with isopyknic infection medium. Mice were scored for disease and weighed every day. The scoring was performed according to the descriptions of other study with some modifications: hair loss (0: none, 1: minimal periocular hair loss, 2: moderate periocular hair loss, 3: severe hair loss limited to periocular, 4: hair loss severe and extensive); hydrocephalus (0: none, 1: minor bump, 2: moderate bump, 3: large bump); symptoms related to neurological disease (0: normal, 1: jumpy, 2: uncoordinated, 3: hunched/lethargic, 4: unresponsive/no movement); eye swell/lesions (0: none, 1: minor swelling, 2: moderate swelling, 3: severe swelling and skin lesions, 4: lesions extensive). The mice were sacrificed when 20% of body weight was lost.

The brain of half mice were fixed with 4% paraformaldehyde (P0099, Beyotime Biotechnology Shanghai, China) for histopathology. P/N/C and cortex of other half brain were acquired and frozen immediately at −80°C. For antibiotics treatment, mice drink water consisting of vancomycin (0.5 g/L, T8641, TargetMol, Boston, MA, USA), ampicillin (1 g/L, T0814L, TargetMol, Boston, MA, USA), neomycin (1 g/L, T0950, TargetMol, Boston, MA, USA), and metronidazole (1 g/L, T1079, TargetMol, Boston, MA, USA) from 5 weeks of age. After 2 weeks, the mice were infected with HSV-1, and the antibiotics were removed until the experimental end point. For the nicotinamide n-oxide treatment, 5-weeks-old were pretreated with antibiotics for 2 weeks, followed by those infected with HSV-1 and treated with NAMO simultaneously.

### 16S rRNA Sequencing

The faces of mice were collected using aseptic 1.5 mL tubes. The genomic DNA of bacteria in fasces was extracted with CTAB/SDS method. 16s rRNA genes of distinct regions were amplified by PCR, and the PCR products were purified with Qiagen Gel Extraction Kit (Qiagen, Germany). Then sequence libraries were generated with TruSeq® DNA PCR-Free Sample Preparation Kit (Illumina, USA). The library quality was assessed on the Qubit@2.0 fluorometer (Thermo Scientific) and sequenced on an Illumination NovaSeq platform and 250 bp paired-end reads were generated.

### Microglia isolation and flow cytometry assay

The microglia of mice was harvested using a percoll gradient followed other study. Briefly, the adult mice killed by cervical dislocation were sterilized with 75% ethanol for 3 min. Subsequently, the brain was collected on plate to mince. The minced brain tissue firstly was filtrated with 100 μm nylon mesh filter and then were centrifuged for 5 min at 1000×g. The sediment was re-suspended in 37% percoll and supplied moderate 70% percoll. After centrifugation, brain cells from strata intermedium were obtained and washed with phosphate buffer saline. Primary antibodies including APC anti-mouse CD11b (101211, Biolegend) and APC/Cy7 anti-mouse CD45 (103116, Biolegend) were added to cells at 4C for 30 min. The microglia were sorted using a BD FACSAria III (Becton Dickinson) and the data were analyzed with FlowJo software.

### RNA extraction and quantitative real-time PCR

Total RNA was extracted with Trizol (Invitrogen, 15596–026, USA) according to the guideline and the concentration was measured using a NanoPhotometer P330 spectrophotometer (IMPLEN, Munich, Germany). 1 μg RNA was reverse transcribed into cDNA by a reagent kit (PrimeScript RT reagent Kit, Takara). Subsequently, the mRNA expression level of target gene was detected by quantitative real-time PCR (qRT-PCR) with a Bio-Rad CFX96 real-time PCR system. The expression of *gapdh* or *β-actin* was used as a reference. The primer sequences were provided in supplementary table 1.

### NAD^+^ and ATP detection

NAD^+^ and NADH were measured with a commercially available NAD^+^/NADH assay kit (Abcam, ab65348) according to the manufacturer’s protocol. BV2 cells (4 × 10^5^) were homogenized in 400 μl lysis buffer and centrifuged at 17,000 g for 5 min at 4°C. Supernatants were filtered using 10 kD filters (Millipore) and spun at 17,000 g for 60 min. To measure NADH, NAD^+^ was decomposed by incubation at 65C for 30 min. Standard curves (5–200 spg/ml) were generated for quantification. ATP levels were measured with a commercially available ATP Assay Kit (Colorimetric/Fluorometric) (Abcam, ab83355) according to the manufacturer’s protocol.

### RNA-sequence

The total RNA of collected microglia and OB tissues was extracted with Trizol reagent, and all mRNA was purified and converted to a cDNA library using NEBNext Ultra II irectional RNA library Prep Kit (Illumina, San Diego, CA, USA). The cDNA library was sequenced on Illumina Hisep X Ten. Quality control checks of raw sequence data were performed by a Fast QC program. For the data analysis, the StringTie-Eb software, Ballgrown software, and the GSEA software were used.

### Untargeted metabolismics

The sera samples of mice were harvested and stored at −80°C until sample analysis. The frozen samples were slowly thawed at 4°C. 400 μl mixed solution of methanol and acetonitrile was added to 100 μl sera sample. Then, the mixture was vortexed, sonicated, and centrifuged for 20 min, to remove protein. All supernatant samples were re-suspended in acetonitrile after being vacuum dried, vortexed and centrifuged again. The collected supernatant were firstly separated by Agilent 1290 Infinity LC. Then isolated samples were divided into two fractions: one was analyzed by ultra-performance liquid chromatography coupled with tandem mass spectrometry (UPLC–MS/MS) with negative ion mode electrospray ionization, the other for analysis by UPLC–MS/MS with positive ion mode electrospray ionization. Finally, the valid data extracted from original data by XCMS procedure were further subjected to a variety of analysis by SIMCA-P software, such as principal component analysis and partial least squares discrimination analysis.

### Isolation of mitochondria

Mitochondria were isolated from BV2 cells using a Mitochondria isolation Kit (89874,Thermo, USA). Briefly, the cells were treated with buffer A, buffer B and buffer C sequentially. Then the nuclei and debris were discarded, the supernatant was collected and centrifuged 12000 × g for 15 min at 4°C, the supernatant after centrifugation was cytosol and the sediment was the mitochondrial pellets. Then the mitochondrial pellets were lysed in RIPA buffer containing protease inhibitors.

### Western Blot

The cells were lysated with SDS buffer (P0013 G, Beyotime, China) containing 1 mM phenylmethylsulfonyl fluoride (PMSF, ST505, Beyotime,) and total proteins were separated by 8%-15% gradient SDS-PAGE(P0688, P0690, P0692, P0695, Beyotime, China). The samples were then transferred to polyvinylidene fluoride (PVDF) membrane (Roche, 03010040001, Mannheim Germany), blocked with 5% skim milk(P0216, Beyotime, China), and incubated with primary antibodies overnight at 4°C. The proteins were then were incubated with appropriate secondary antibodies for 1 h. Target proteins were detected with ECL solutions (P0018FS, Beyotime, China) by Gel Imaging System (Bio-Rad, Hercules, CA, USA). The band intensity was calculated using Image J software and was normalized to the level of GAPDH or β-actin.

### Histology

Tissues fixed in 4% paraformaldehyde were embedded in paraffin and cut into slices. For hematoxylin-eosin (H&E) staining, the samples were dewaxed and stained with hematoxylin and eosin. Images were captured with a light microscope. For immunohistochemistry (IHC), after dewaxing and heat-induced antigen retrieval, the tissue slices were subjected to incubation with anti-VP5 (HSV-1 capsid protein, ab6508, Abcam) or anti-Iba-1 (10904-1-AP, Proteintech) antibodies at 4°C overnight. The samples were then incubated with appreciate second antibodies (7076, 7074, CST), followed by coloration with dibenzothiophene and stained with hematoxylin. After a series of experiments, the brain slices were detected and analyzed with a light microscope.

### Immunofluorescence assay

BV2 cells inoculated in confocal dishes were treated with HSV-1 and NAMO for indicated time, the cells were then fixed with 4% paraformaldehyde, permeabilized with 0.1% Triton X-100 and blocked with 5% bovine serum albumin. Subsequently, the cells were incubated with p65 antibody overnight at 4°C and were treated with Alexa Fluor 488 (green)-labeled secondary antibody (A32790, Thermo Fisher Scientific) for 1 h at room temperature. The nucleus were stained with DAPI (C1006, Beyotime, China). Finally, fluorescent images were obtained by a confocal laser scan microscope (LSM 510 meta, Zeiss). For mitophagy assay, BV2 cells were transfected with GFP-LC3B (2 μg) or mitoKeima (1 μg) plasmids using Lipofectamine 3000 transfection reagent (ThermoFisher, L3000015) for 24 h before infected with HSV-1 and treated with NAMO for another 12 h. The cells transfected with GFP-LC3B plasmids were fixed and stained with Mito-Tracker Red (ThermoFisher, M7512) for 0.5 h. The cells transfected with mitoKeima plasmids were untreated. Finally, fluorescent images were obtained by a confocal laser scan microscope (LSM 510 meta, Zeiss). For in vivo imaging, normal or ABX-treated mice were infected with EGFP-HSV-1, and the in vivo EGFP distribution image was taken with the Bruker Small Animal Optical Imaging System (In‐Vivo Xtreme II; Billerica, MA).

### Bacteria culture

*Lactobacillus reuteri* (GDMCC 1.614), *Lactobacillus garneri* (GDMCC 1.984) and *Bacteroides sartorii* (GDMCC 1.1541) were purchased from Guangdong microbial culture collection center. The bacteria were cultured in Gifu Anaerobic Medium under anaerobic conditions manufactured by AnaeroPack-Anaero (Mitsubishi Gas Chemical, Tokyo, Japan). *L. reuteri, L. garneri* and *B. satorii* were incubated with nicotinamide (100 μM or 200 μM) for 12 h or 24 h. The supernatant was then harvested after centrifugation, and the precipitates of bacteria were reconstituted in medium, crushed and centrifuged. The supernatant was then mixed with acetonitrile, vortexed for 3 min and followed by centrifugation at 13000 × g for 15 min to extract nicotinamide and its metabolisms. All the supernatant was harvested and dried using gas pressure blowing concentrator. Then the residue was resuspended in 100 μL of water/methanol (50:50, V/V), followed by centrifugation at 13000 × g for 15 min. Finally, the samples were analyzed by the UPLC-QTOF/MS system.

### Ultrahigh-performance liquid chromatography equipped with quadrupole time-of-flight mass spectrometry (UPLC-QTOF/MS) analysis

Nicotinamide and niacinamide nitrogen oxide were quantified using a Waters UPLC-QTOF/MS system, which is composed of ACQUITY UPLC and a Xevo G2 QTOF mass spectrometer. Chromatographic separation was performed using a BEH Amide column (1.7 μm, 2.1 × 100 mm) (Waters, Milford, MA). The phase A of the mobile phase is 20 mM ammonium acetate in water, and the phase B is acetonitrile in 0.1% formic acid. The flow rate was 0.2 ml/min. The gradient elution program was 5% phase B for 0–1 min, 5–10% phase B for 1–2 min, 10–50% phase B for 2–3.5 min, 50–95% phase B for 3.5–4 min, 95% phase B for 4–4.5 min, and 95–5% phase B for 4.5–5 min. The mass spectrometer was operated at the positive ion mode.

### Statistical analysis

Data were presented as mean ± SD of the results from at least 3 independent experiments. Data were analyzed by one-way analysis of variance or Student’s t test as appropriate, with significance set as p < .05 (*), p < .01 (**) or p < .001 (***).

## Supplementary Material

Supplemental Material

## Data Availability

All data generated in this study are included in this article and supplementary information or are available from the corresponding author on reasonable request due to an ongoing collaboration with multiple principal investigators. Other sequence data of the original study are available in https://pan.baidu.com/s/12RNDoxKjz0qfD4xeESh3Uw (Extraction code: 2022)
