# Supplementary material for: The intestinal microbial metabolite nicotinamide n-oxide prevents herpes simplex encephalitis via activating mitophagy in microglia
Source: Gut Microbes. 2022 Jul 6;14(1):2096989. doi: 10.1080/19490976.2022.2096989 (PMC9262364; doi:10.1080/19490976.2022.2096989)
Supplement: Supplemental Material [file KGMI_A_2096989_SM3814.doc]

**Supplementary information**

**The microbial metabolite nicotinamide n-oxide protects mice from herpes simplex encephalitis**

Feng Li, Yiliang Wang, Xiaowei Song, Zhaoyang Wang, Jiaoyan Jia, Shurong Qing, Lianzhou Huang, Yuan Wang, Shuai Wang, Zhe Ren, Kai Zheng, Yifei Wang

**Supplementary materials and methods**

**Viral inactivation, attachment and penetration assay**

For viral attachment assay, pre-cooled N2A cells were incubated with HSV-1 (50 PFUs/well) and different concentrations of NAMO at 4 °C for 2 h to allow the attachment of viral particles. The virus inoculum was then removed and the cells were washed and replenished with a cover layer. After 24 h incubation at 37 °C, total viral DNA was extracted and the *UL27* DNA level was analyzed by qRT-PCR. For viral penetration assay, pre-cooled N2A cells were infected with HSV-1 at 4 °C for 2 h. The culture medium was then replaced with medium containing NAMO, and the cells were incubated at 37 °C for 10 min. After incubation, PBS buffer (pH = 3) was added to wash the cells three times, and alkaline PBS (pH=11.0) was added for 1 min to remove the bound but not entered virions. Then the cells were replenished with a cover layer and incubated at 37 °C for 24 h to detect *UL27* DNA level.

**Supplementary table 1 Primer sequence used in qRT-PCR**

| Primers | Forward (5’-3’) | Reverse (5’-3’) |
| --- | --- | --- |
| Tnf-α | CATCTTCTCAAAATTCGAGTGACAA | TGGGAGTAGACAAGGTACAACCC |
| Il-6 | CTGCAAGAGACTTCCATCCAG | AGTGGTATAGACAGGTCTGTTGG |
| Nos2 | CAGAGGACCCAGAGACAAGC | TGCTGAAACATTTCCTGTGC |
| Icp0 | CCCACTATCAGGTACACCAGCTT | CTGCGCTGCGACACCTT |
| *β-actin* | GTGACGTTGACATCCGTAAAGA | GCCGGACTCATCGTACTCC |
| *Gapdh* | AGGTCGGTGTGAACGGATTTG | GGGGTCGTTGATGGCAACA |

**Supplementary Figures and legends**

**
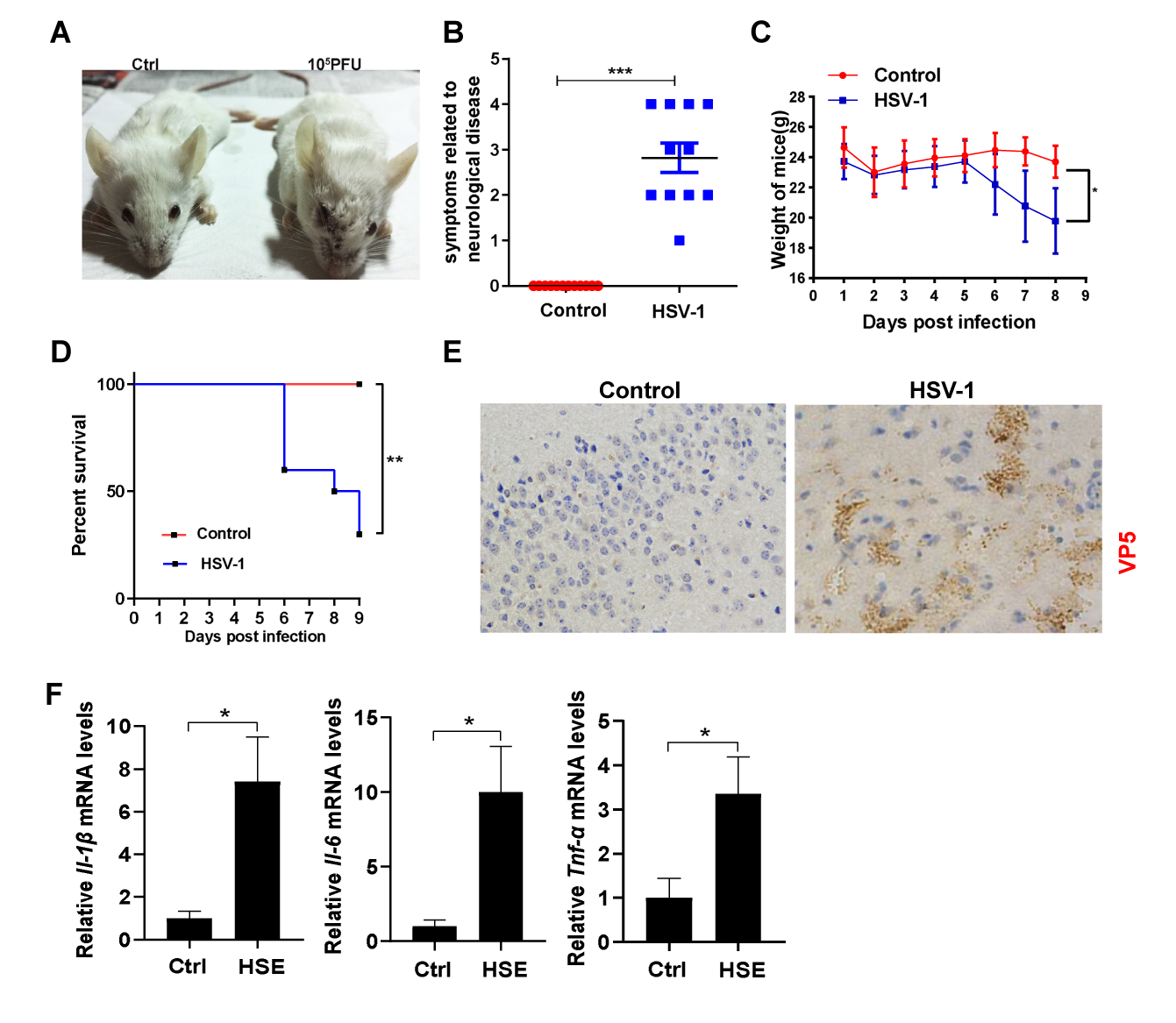
**

**Figure S1. The HSE model with intranasal HSV-1 infection. (A)** Representative photograph of HSE mice or normal mice (control). **(B)** Mice were scored for HSE symptoms related to neurological disease. Each symbol represents data from one mice (n=8-12 mice per group). Data are mean±SD. Significant differences were determined by an unpaired t test (*p < 0.05, **p < 0.01, ***p < 0.001). **(C-D)** The weight (C) and survival of HSE and normal mice (D). *p < 0.05 versus ctrl group. **(E)** The brain regions were stained with immunohistochemistry for VP5 to detect HSV-1. (**F**) qRT-PCR analysis of the intestinal inflammation of HSE mice. Total RNA of intestinal tissues derived from HSE mice was extracted and the mRNA expression levels of TNFα, IL-6 and IL-1β were analyzed by qRT-PCR, respectively. ***p < 0.001 versus control group.

**
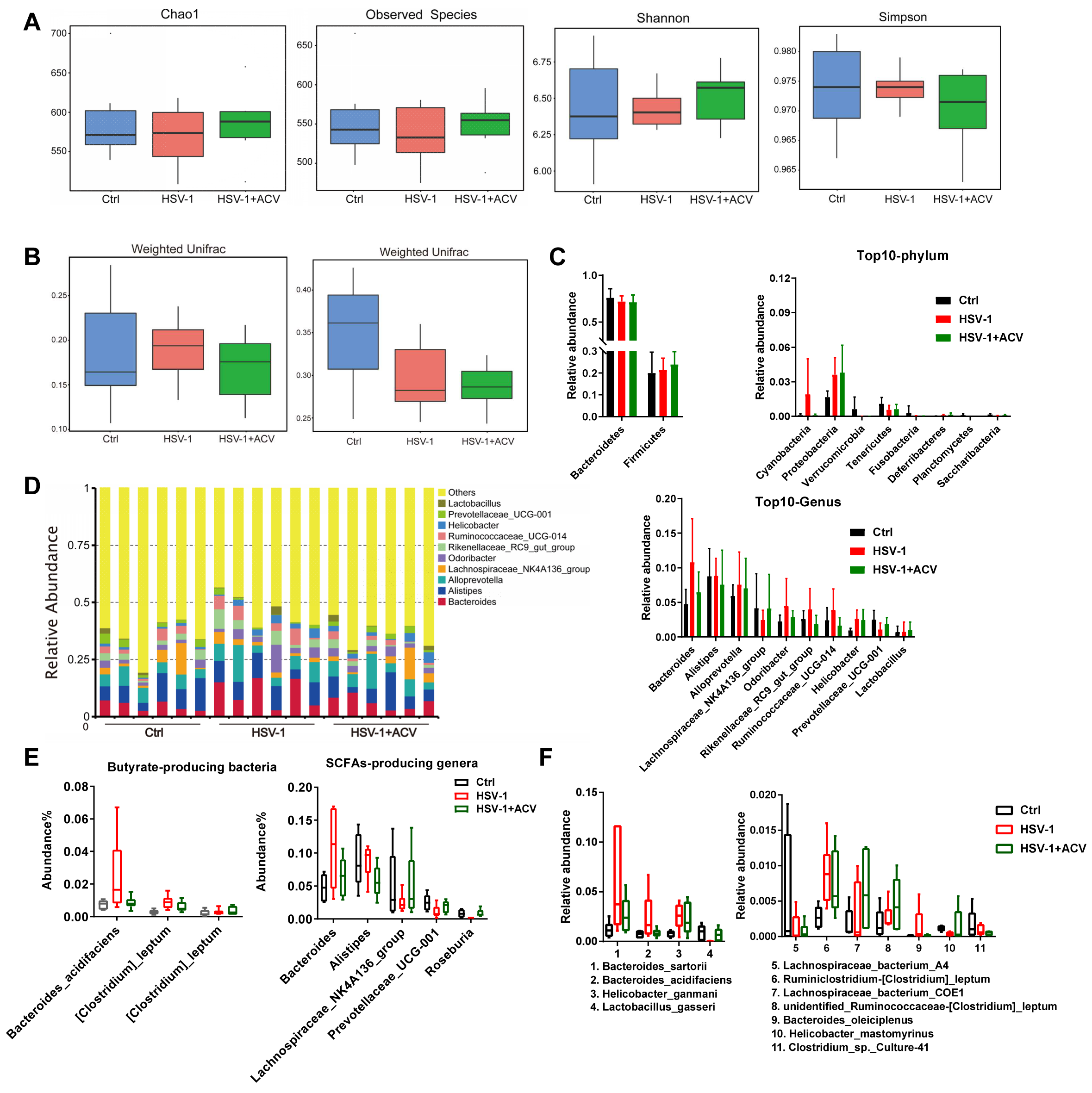
**

**Figure S2. Changes in microbes upon HSV-1 infection and/or ACV treatment. (A-B)** Measures of alpha diversity (chaos, observed species, shannon, and simpson) (A), or beta diversity (weighted_unifrac and unweighted_unifrac) showed bacterial diversity among the Ctrl, HSV-1 and HSV-1 + ACV group. **(C-D)** Top10 microbes at the phylum (C) and genus levels (D) among the Ctrl, HSV-1 and HSV-1 + ACV groups. **(E)** Relative abundance of butyrate-producing and SCFAs-producing genera in Ctrl, HSV-1, and HSV-1 + ACV groups. Data are mean±SD (n=8-10). **(F)** The most different microbes at the species level among Ctrl, HSV-1 and HSV-1 + ACV group. Data are mean±SD (n=8-10).


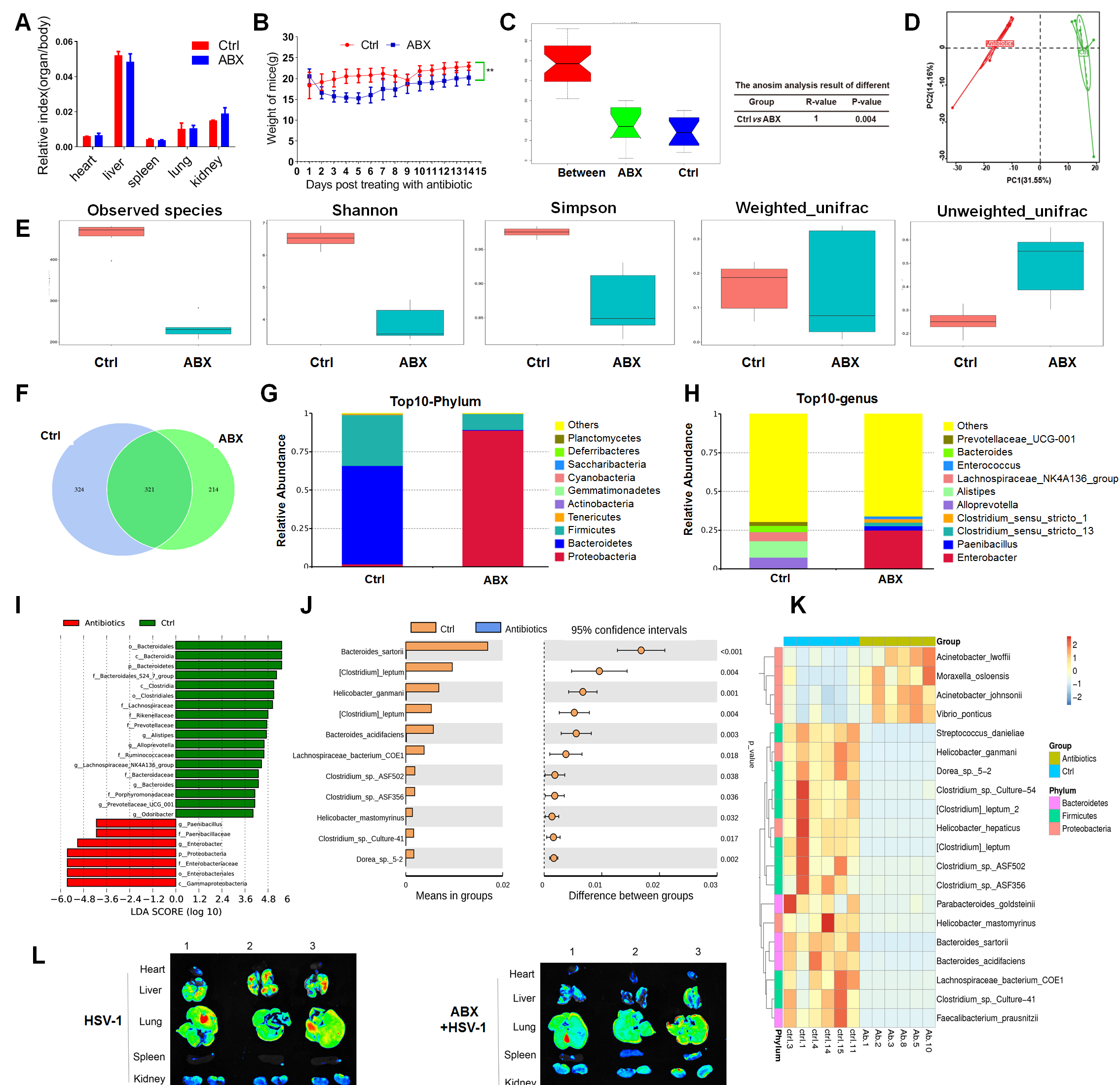


**Figure S3. Antibiotics treatment depletes gut microbes. (A)** The relative index (organ/body) in heart, liver, spleen, lung and kidney from antibiotic (ABX)-treated and ctrl mice, respectively. **(B)** A 15 days recording in mouse weight of ABX-treated and ctrl mice. n=6 mice per group. **(C-D)** PCoA analysis (C) or ANOSIM analysis (D) of gut bacterial communities between ABX-treated and ctrl mice. **(E)** Measures of alpha diversity (observed species, shannon, simpson), or beta diversity (weighted_unifrac and unweighted_unifrac) showed bacterial diversity between ABX-treated and ctrl mice. **(F)** Venn diagram showed the overlapped OTUs between ABX-treated and ctrl mice. **(G-H)** Top10 microbes at the genus level (G) or at the phylum level (H) between ABX-treated and ctrl mice. **(I)** Graphics of Linear discriminant analysis (LDA) effect size showed microbes in genera level between ABX-treated and ctrl mice. Horizontal bars represent the effect size for each taxon. The length of the bar represents the log10 transformed LDA score, indicated by vertical dotted lines. **(J)** Difference of bacteria in species levels between ABX-treated and ctrl mice. **(K)** Heatmap for Top20 microbes in ABX-treated and ctrl mice (n=6 mice). Red indicates high expression, and blue indicates low expression, as shown in the scale bar. (**L**) Distribution of EGFP-HSV-1 in heart, liver, spleen, lung and kidney between ABX-treated and ctrl mice. Representative graphs were shown.


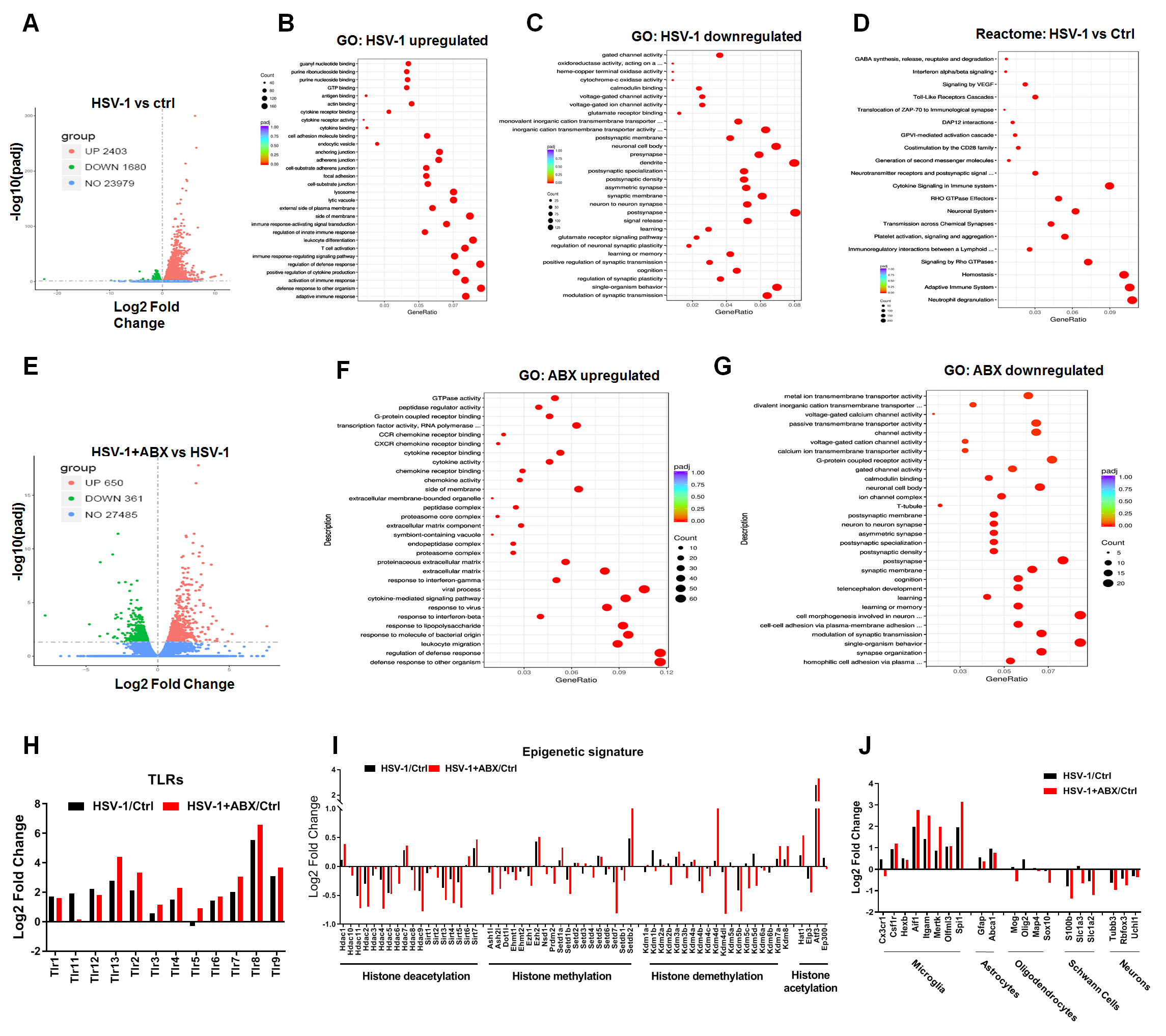


**Figure S4. Gut microbe depletion alters immune-related signaling pathway activation and gene expression. (A)** Volcano plot of differentially expressed genes (DEGs) in OB derived from ABX-treated and ctrl mice. Fold change and the adjusted p-value were shown. **(B-C)** GO pathway analysis of up-regulated DEGs (B) or downregulated DEGs (C) by HSV-1 infection in OB. **(D)** Reactome pathway analysis of DEGs enriched in key cellular processes in OB between HSV-1 and Ctrl group. **(E)** Volcano plot of DEGs between HSV-1 and HSV-1 + ABX group. **(F-G)** GO pathway analysis of up-regulated DEGs (F) or downregulated DEGs (G) by ABX treatment in OB when compared with HSV-1 group. **(H-J)** mRNA expression values (log2Fold change) of genes from OB in Ctrl, HSV-1 and HSV-1 +ABX mice. Expression of TLRs (H) and the central transcriptional and survival factors of different CNS cell types (J) were presented. Epigenetic signature (I) were examined by investigating genes (log2 Fold change) related to histone acetylation, deacetylation, methylation and demethylation when compared to ctrl group, respectively. Bars represent mean±SD with 6 samples in each group.


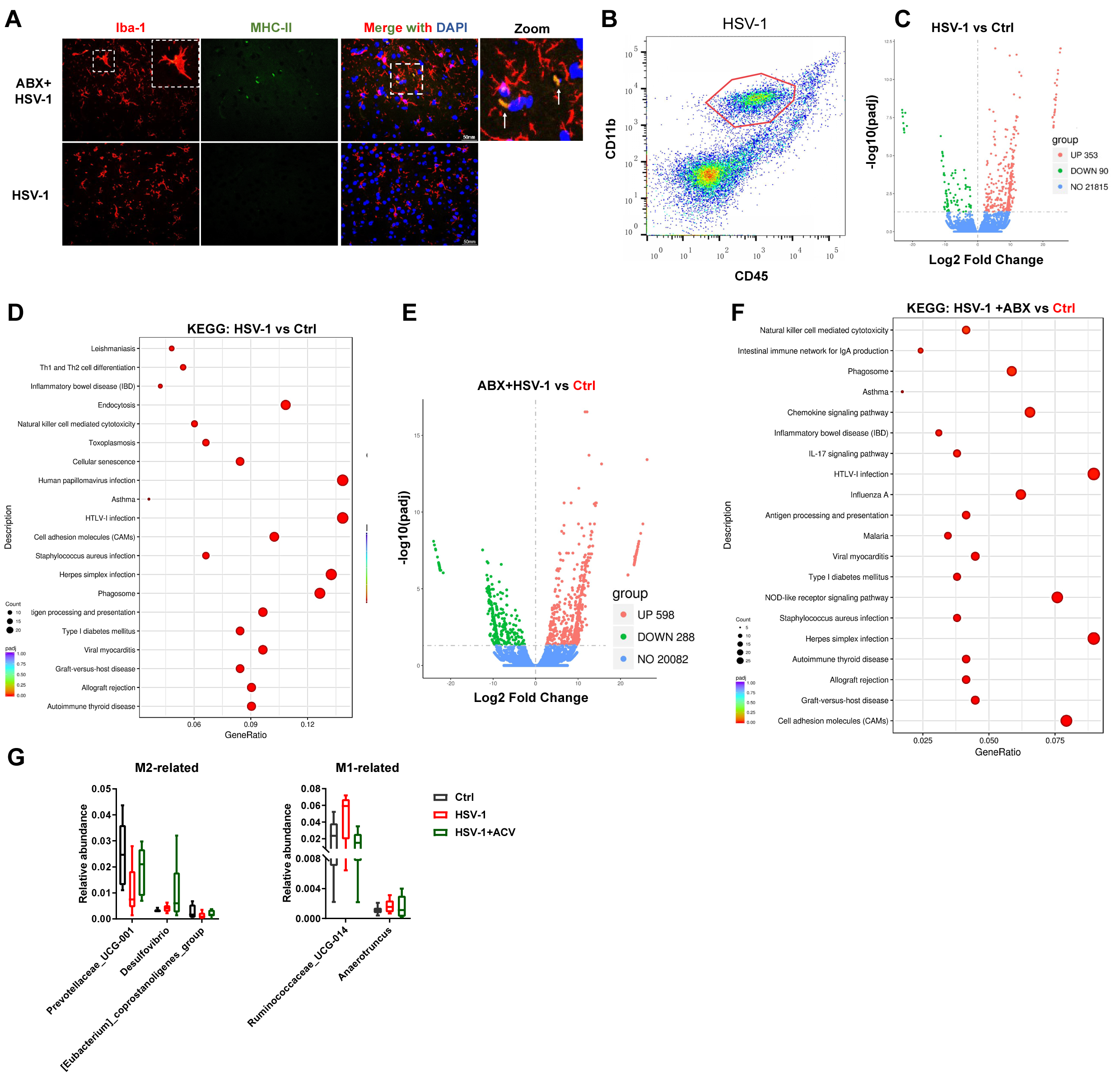


**Figure S5. Lack of microbes enhances immune response in microglia. (A)** Immunofluorescence of Iba-1 and MHC-II in OB derived from ABX-treated and ctrl mice. OB tissues were fixed and sequentially stained with anti-Iba-1 and anti-MHC-II antibodies. Scale bar, 10 μm. **(B)** Isolation of CD11b^+^ CD45^lo^ microglia by flow cytometry. **(C-D)** Volcano plot (C) or KEGG pathway (D) of DEGs in isolated microglia derived from HSV-1 infected and ctrl mice. **(E-F)** Volcano plot (E) or KEGG pathway analysis (F) of DEGs in sorted microglia derived from HSV-1 infected mice with or without ABX treatment. **(G)** Relative abundances of M1-related and M2-related bacteria derived from Ctrl, HSV-1 and ABX + HSV-1 mice. Bars represent mean±SD with 6 samples in each group.


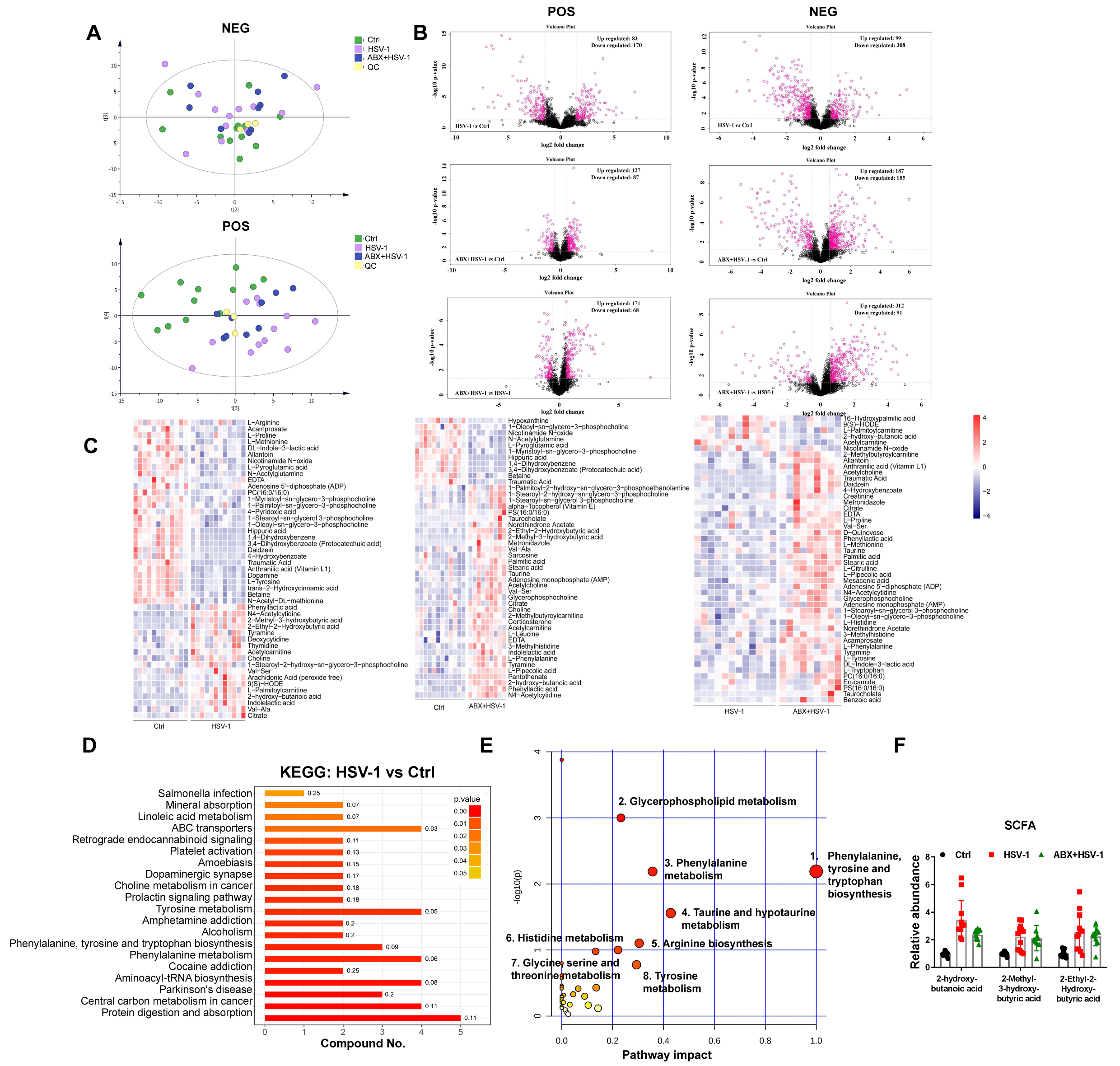


**Figure S6. HSV-1 infection and antibiotics treatment alters serum metabolite composition. (A)** PCA analysis of serum samples from Ctrl, HSV-1, and ABX + HSV-1 mice in positive (POS) and negative (NEG) ion mode of mass spectrometric analysis, respectively. **(B)** Volcano plot of serum metabolites in HSV-1 vs Ctrl, ABX + HSV-1 vs Ctrl, and ABX + HSV-1 vs HSV-1 in POS and NEG ion mode of mass spectrometric analysis, respectively. **(C)** Heatmap of differentially expressed metabolites from HSV-1 vs Ctrl, ABX + HSV-1 vs Ctrl, and ABX + HSV-1 vs HSV-1. Red indicates high expression and blue indicates low expression of metabolites as shown in the scale bar. **(D)** KEGG pathway analysis of differentially expressed metabolites in serum between HSV-1 and Ctrl group. **(E)** Metabolite set enrichment analysis. *x* axis represents the pathway impact, and *y* axis represents the pathway enrichment. Larger sizes and darker colors represent increased pathway enrichment and higher pathway impact values, respectively. (F) Relative abundances of SCFAs in serum derived from Ctrl, HSV-1, and ABX + HSV-1 mice, respectively. Data are mean±SD (n=8-12).


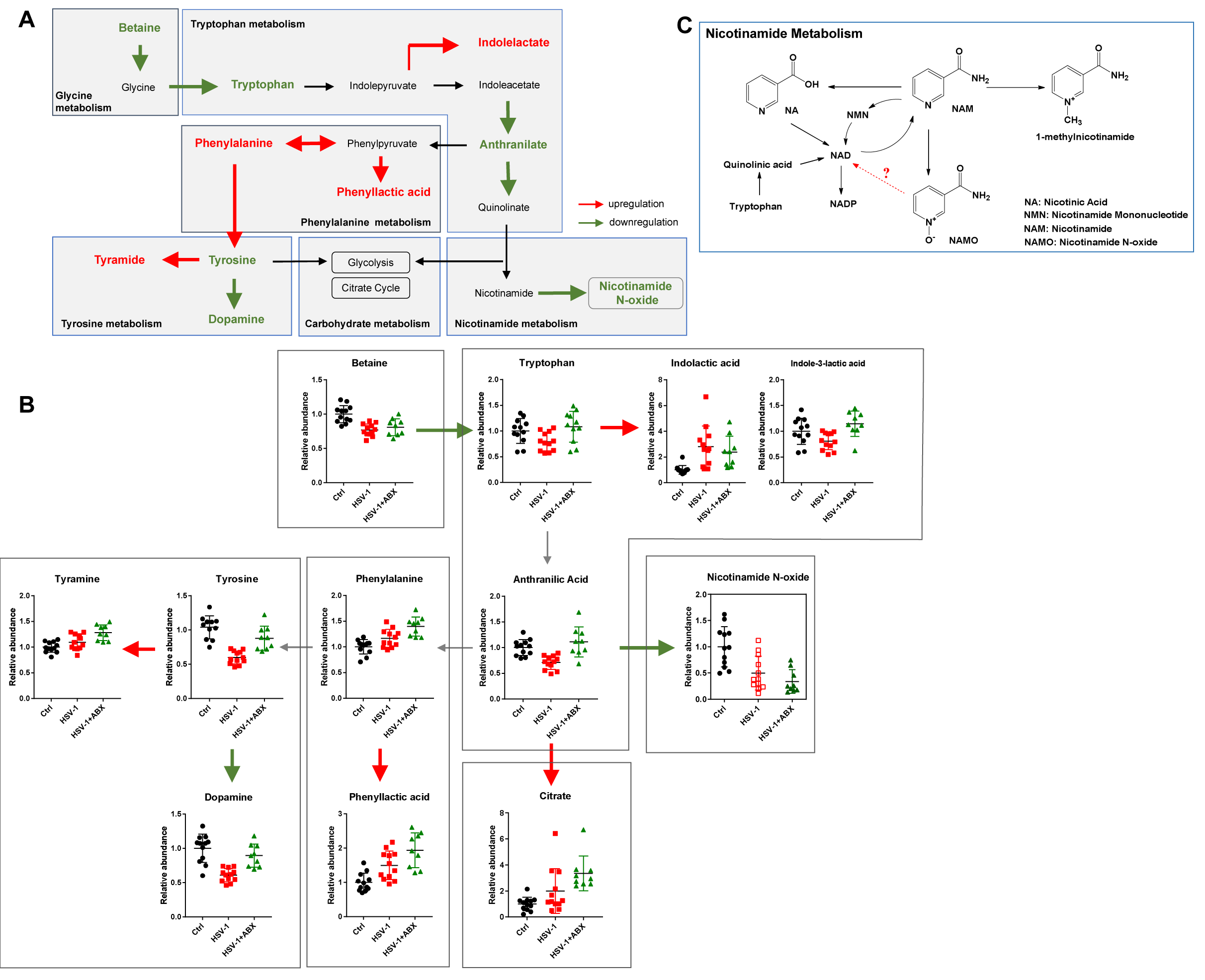


**Figure S7. HSV-1 infection and ABX treatment alters amino acid metabolism. (A)** A schematic diagram shows the interaction between glycine, tryptophan, phenylalanine, tyrosine and nicotinamide (NAM) metabolism pathways. Red arrow indicates ABX-mediated upregulation and green arrow indicates ABX-mediated downregulation when compared with HSV-1 group. **(B)** Relative abundances of metabolites enriched in glycine, tryptophan, phenylalanine, tyrosine and NAM metabolisms. Data are mean±SD (n=8-12). **(C)** A schematic diagram of NAM metabolism pathway.


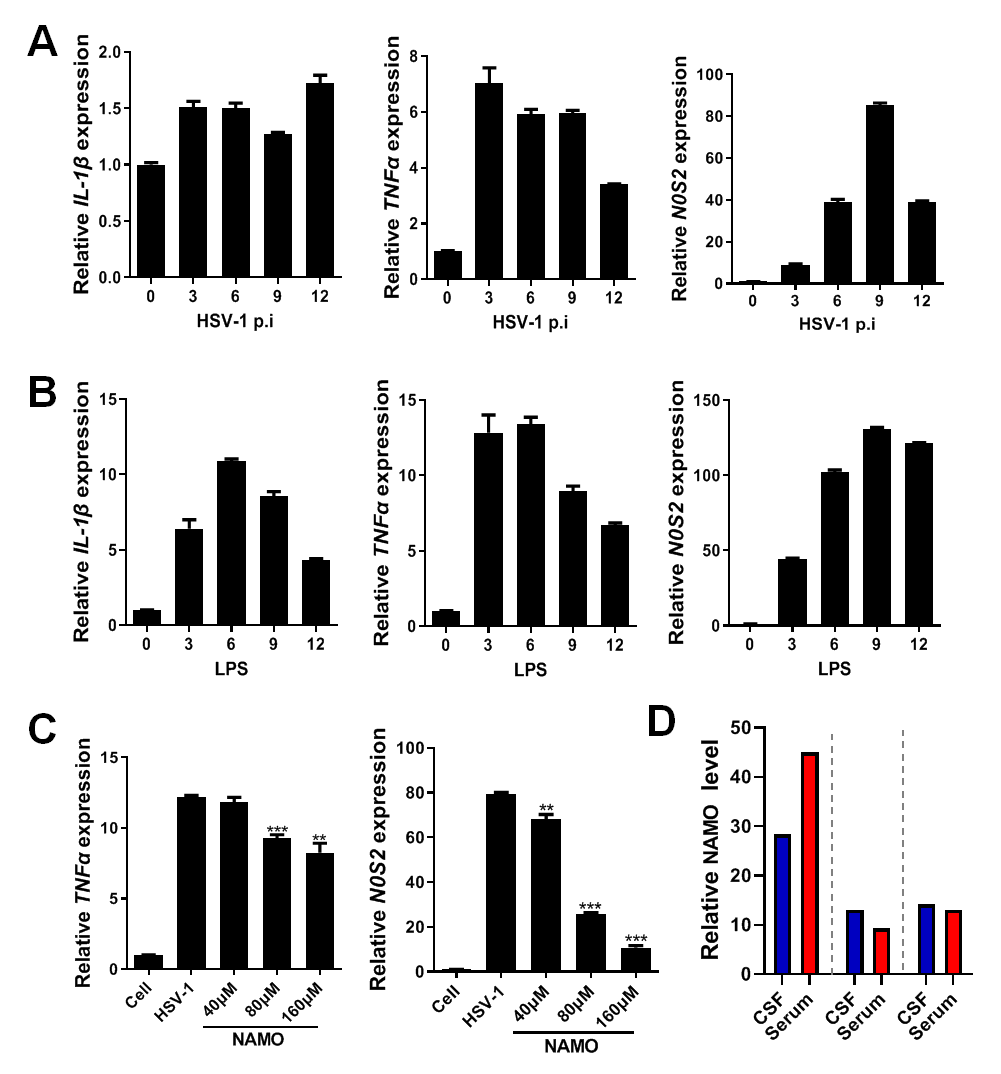


**Figure S8. NAMO inhibits HSV-1-induced inflammation in microglia. (A-B)** The mRNA expression of *TNF-α,* *IL-1β* and *NOS2* in BV2 cells at 0, 3, 6, 9, and 12 hours post HSV-1 infection (MOI=1) (A) or LPS treatment (B) was detected by qRT-PCR. Data are mean±SD from three independent experiments. **(C)** BV2 cells were infected with HSV-1 (MOI=1) in the presence of NAMO for 3 h and total RNA was extracted to detect the mRNA expression of *TNF-α* and *NOS2* via qRT-PCR. Significant differences were determined by an unpaired t test (*p < 0.05, **p < 0.01, ***p < 0.001). **(D)** Relative NAMO levels in CSF or serum derived from mice treated with NAMO for 4 h (n=3).

**
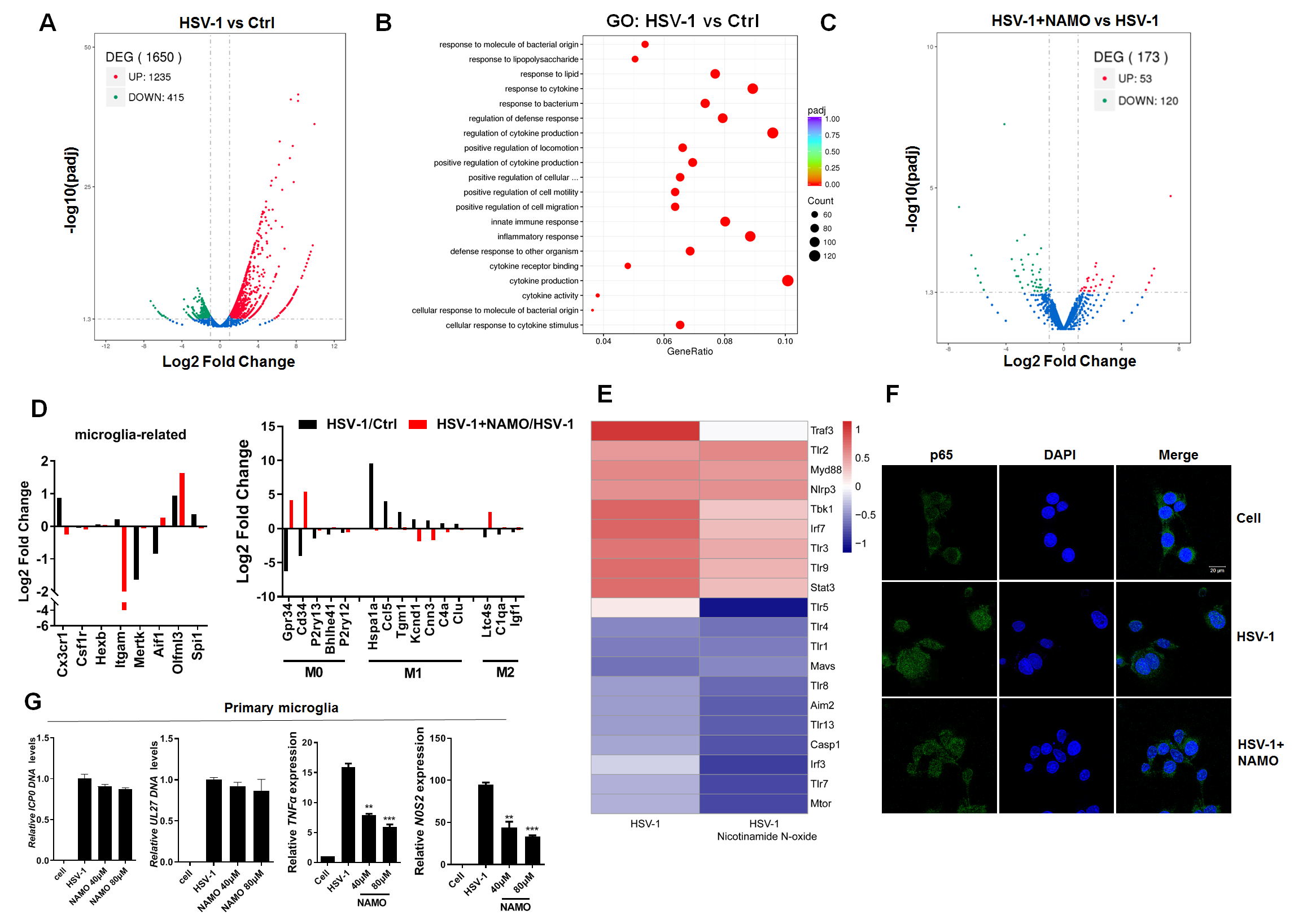
**

**Figure S9. NAMO inhibits microglia activation. (A)** Volcano plot of differential expressed genes (DEGs) between HSV-1-infected and mock-infected BV2 cells. **(B)** GO pathway analysis of DEGs in BV2 cells between HSV-1 and Ctrl group. **(C)** Volcano plot of DEGs between HSV-1+NAMO and HSV-1 group. **(D)** mRNA expression values (log2Fold change) of central microglia transcripts and survival factors (left panel), as well as microglia M0/M1/M2 phenotypes related genes (right panel) in HSV-1 infected cells compared to control and in NAMO-treated cells compared to HSV-1. **(E)** Heatmap for DEGs involved in innate antiviral immune response in HSV-1-infected cells treated with or without NAMO. Red indicates higher expression and blue indicates lower expression as shown in the scale bar. **(F)** Representative immunofluorescent images. BV2 cells were infected with HSV-1 (MOI=1) in the presence or absence of NAMO (40 μM) for 12 h and were then fixed, stained with p65 (green) and DAPI (blue). Scale bar, 20 μm. (**G**) Primary microglia isolated by flow cytometry were infected with HSV-1 (MOI=1) in the presence of NAMO for 24 h. Virus replication were examined by measuring *ICP0* and *UL27* DNA levels. Cells were treated with HSV-1 and NAMO for 3 h to analyze the mRNA expression of *TNF-α* and *NOS2* via qRT-PCR. Significant differences were determined by an unpaired t test (*p < 0.05, **p < 0.01, ***p < 0.001).


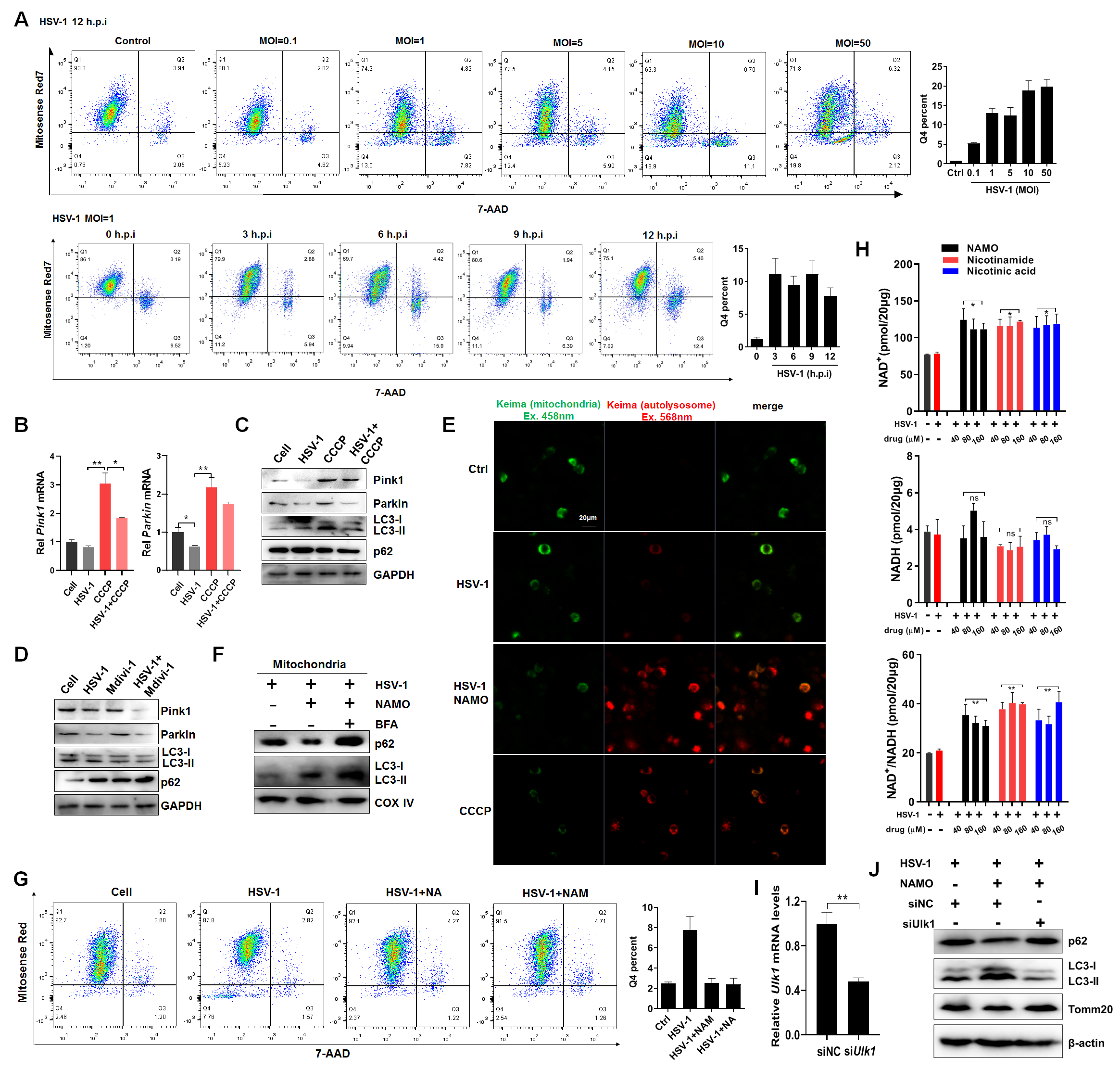


**Figure S10. NAMO ameliorates HSV-1 induced mitochondrion damage. (A)** Mitochondrial membrane potential measurement by flow cytometry. BV2 cells were infected with HSV-1 at different MOIs (0.5, 1, 5, 10 and 50) for 12 h, or infected with HSV-1 (MOI=1) at different times (0, 3, 6, 9, and 12 h.p.i). The percentage (%) of negatively labelled Mitosense Red7/7-AAD were depicted. Data are mean±SD from three independent experiments. (**B-D**) BV2 cells were treated with CCCP (10 μM) (B, C) and Mdivi-1 (20 μM) (D) with or without the infection of HSV-1 for 12 h. Total RNA or protein was extracted and the expression levels of Pink1/Parkin were determined by RT-PCR or WB, respectively. (**E**) BV2 cells were transfected with mito-Keima plasmids (1 μg) for 36 h before HSV-1 (MOI=1) infection and treatment with NAMO (40 μM) or CCCP (10 μM) for 12 h. Fluorescent images were captured by a confocal microscopy. (**F**) BV2 cells were infected with HSV-1 (MOI=1) in the presence or absence of NAMO (40 μM) or BFA (10 μM) at 12 h and mitochondria protein was extracted and subjected to western blot assay. **(G)** Analysis of mitochondrial membrane potential of BV2 cells in Ctrl, HSV-1, HSV-1 + NA (40 μM) and HSV-1+NAM (40 μM) groups. **(H)** BV2 cells were infected with HSV-1 in the presence of NA or NAM for 12 h, and relative NAD+, NADH and NAD+/NADH levels were determined. Data are mean±SD (n=3 independent experiments), with the significance as *p < 0.05, **p < 0.01, ***p < 0.001 versus HSV-1 group. (**I**) The mRNA level of *Ulk1* in the presence of siNC or siULK1. Data are mean±SD, **p < 0.01 versus siNC group. (**J**) BV2 cells were transfected with siNC or siULK1 for 24 h, then the cells were infected with HSV-1 (MOI=1) in the presence or absence of NAMO (40 μM) for 12 h and total protein was subjected to western blot assay.

**
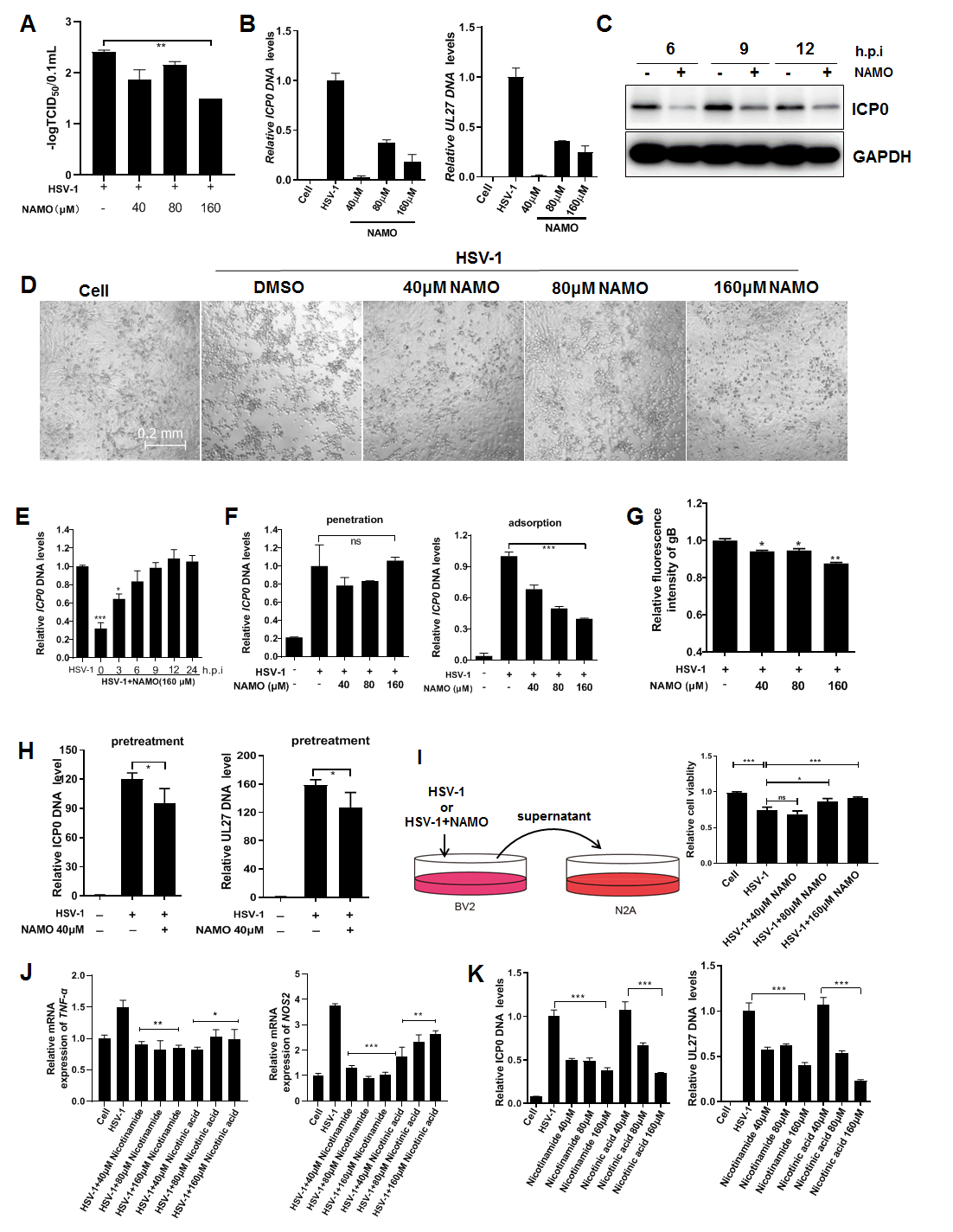
**

**Figure S11. NAMO prevents HSV-1 infection in neuronal cells. (A-B)** Neuronal N2A cells were infected with HSV-1 (MOI=1) in the presence of NAMO for 24 h. Virus titers (A) and virus replication (B) (by measuring ICP0 and *UL27* DNA levels) were examined. **(C)** N2A cells were infected with HSV-1 in the presence of NAMO (40 μM) for 6, 9, or 12 h, and western blot assay was performed to detect viral protein ICP0. **(D)** Morphological changes of N2A cells treated with HSV-1 and NAMO. **(E)** Time-of-addition experiments. HSV-1-infected N2A cells were treated with NAMO at indicated times and the DNA levels of viral *ICP0* were examined by DNA-based qRT-PCR. **(F)** Effects of NAMO on viral penetration and absorption (by measuring *UL27* DNA levels) in N2A cells infected with HSV-1 (see supplementary methods). **(G)** Flow cytometry analysis. Pre-cooled N2A cells were incubated with HSV-1 (MOI=5) and different concentrations of NAMO at 4 °C for 2 h to allow the attachment of viral particles. Total cells were collected and the fluorescent intensity of gB on cell membrane was analyzed by flow cytometry. **(H)** N2A cells were pretreated with NAMO for 2 h. The cells were then infected with HSV-1 for 12 h and viral *ICP0* DNA levels were examined. **(I)** Left panel: schematic diagram depicting the effect of conditional medium derived from HSV-1-infected BV2 cells on N2A cells. Right panel: N2A cells were treated with conditional medium derived from HSV-1 infected BV2 cells with or without NAMO for 24 h and cell viability was examined. **(J)** Effects of Nicotinamide and Nicotinic acid on mRNA expression of *TNF-α* and *NOS2* in BV2 cells infected with HSV-1. **(K)** Effects of Nicotinamide and Nicotinic acid on *ICP0* and *UL27* DNA levels in N2A cells infected with HSV-1. Data are mean±SD (n=3 independent experiments), with the significance as *p < 0.05, **p < 0.01, ***p < 0.001 versus HSV-1 group.


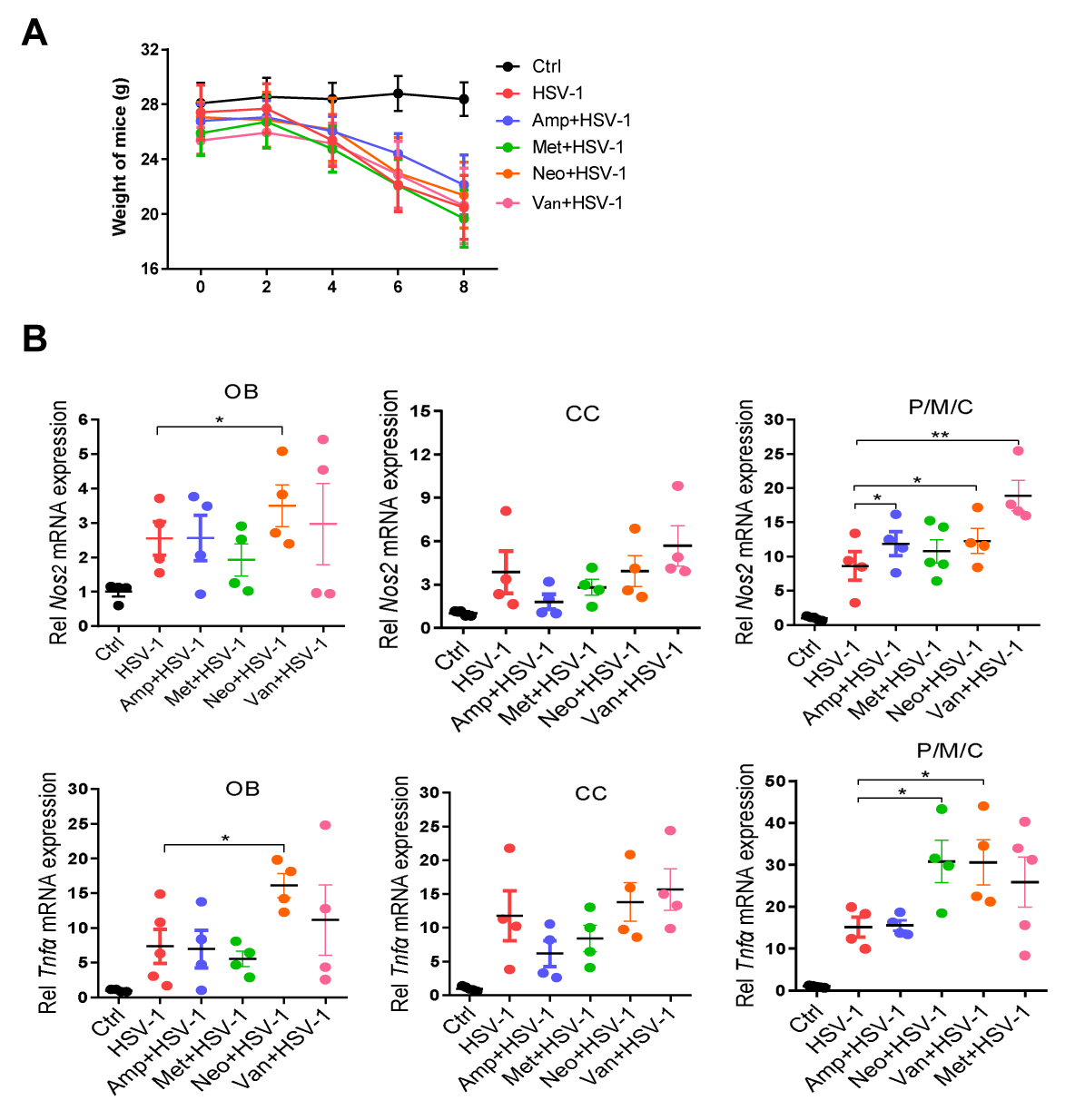


**Figure S12. Single antibiotic treatment aggregates HSE pathology. (A)** Effects of neomycin (Neo), metronidazole (Metro), vancomycin (Vanco), and Ampicillin (Amp) on the body weight of mice infected with HSV-1. Data are mean±SD**.** n=6-8 mice per group. **(B-D)** Effects of single antibiotic on the mRNA expression of *TNF-α* and *NOS2* in olfactory bulb (OB) (B), cerebral cortex (CC)(C), and pons/medulla oblongata/cerebellum (P/M/C) (D) derived from mice infected with HSV-1. Data are mean±SD**.** n=6-8 mice per group. Significance set as *p < 0.05, **p < 0.01, ***p < 0.001 versus HSV-1 group.


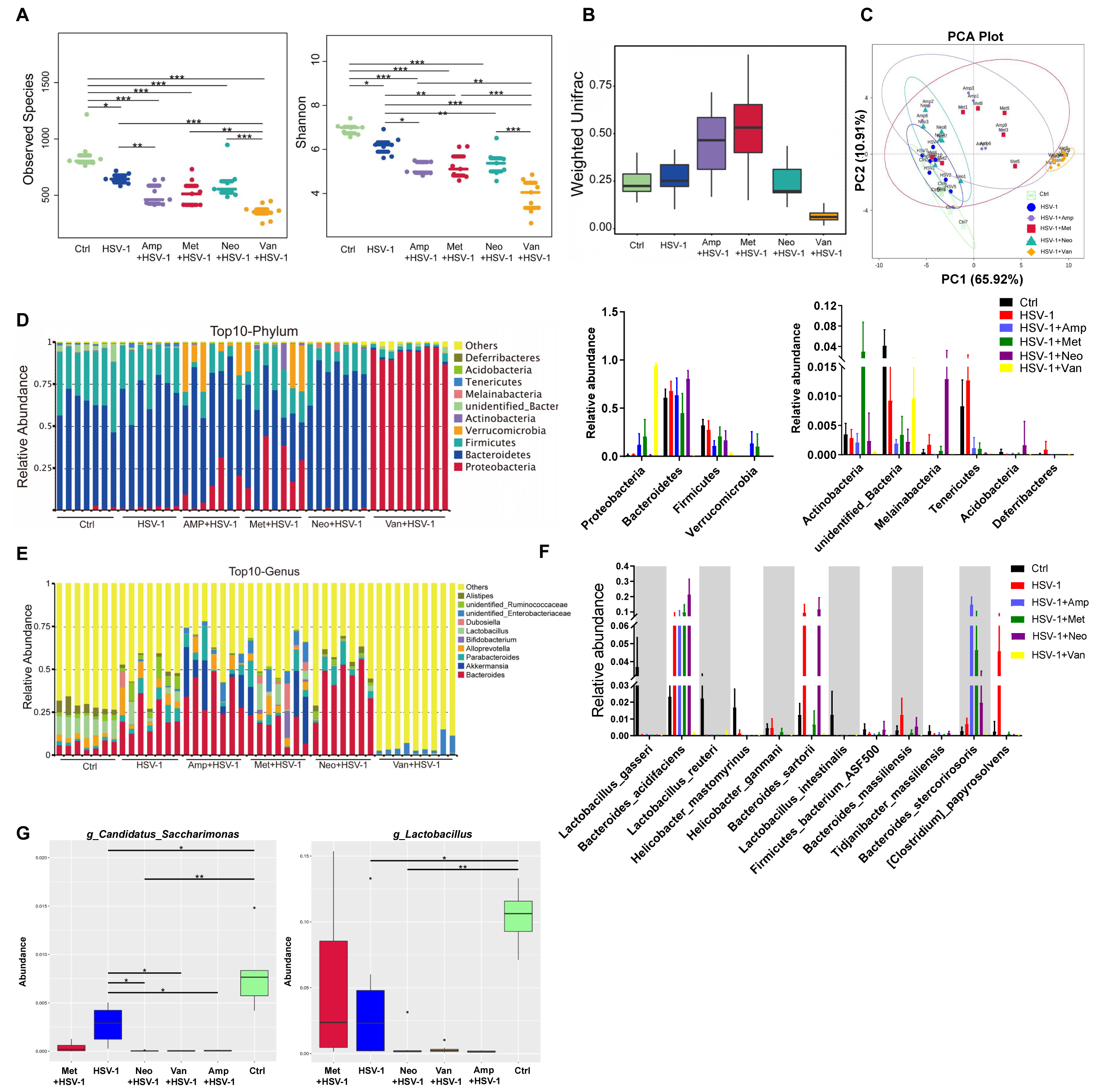


**Figure S13. Gut microbial changes upon HSV-1 infection and single antibiotic treatment. (A-B)** Measurements of bacterial alpha diversity (observed species and Shannon) (A), or beta diversity (weighted_unifrac) in faecal samples from the Ctrl, HSV-1, HSV-1 + Neo, HSV-1 + Met, HSV-1 + Van and HSV-1 + Amp groups. **(C)** PCoA analysis of gut bacterial communities. **(D-E)** Top10 microbes at the phylum (D) or genus level (E). **(F)** Relative abundance of the most differentially expressed 12 bacterial species. **(G)** Bacterial genus whose levels were significantly downregulated by HSV-1 but further reduced by neomycin. n=6-8 mice per group. Significance set as *p < 0.05, **p < 0.01, ***p < 0.001 versus HSV-1 group.

**
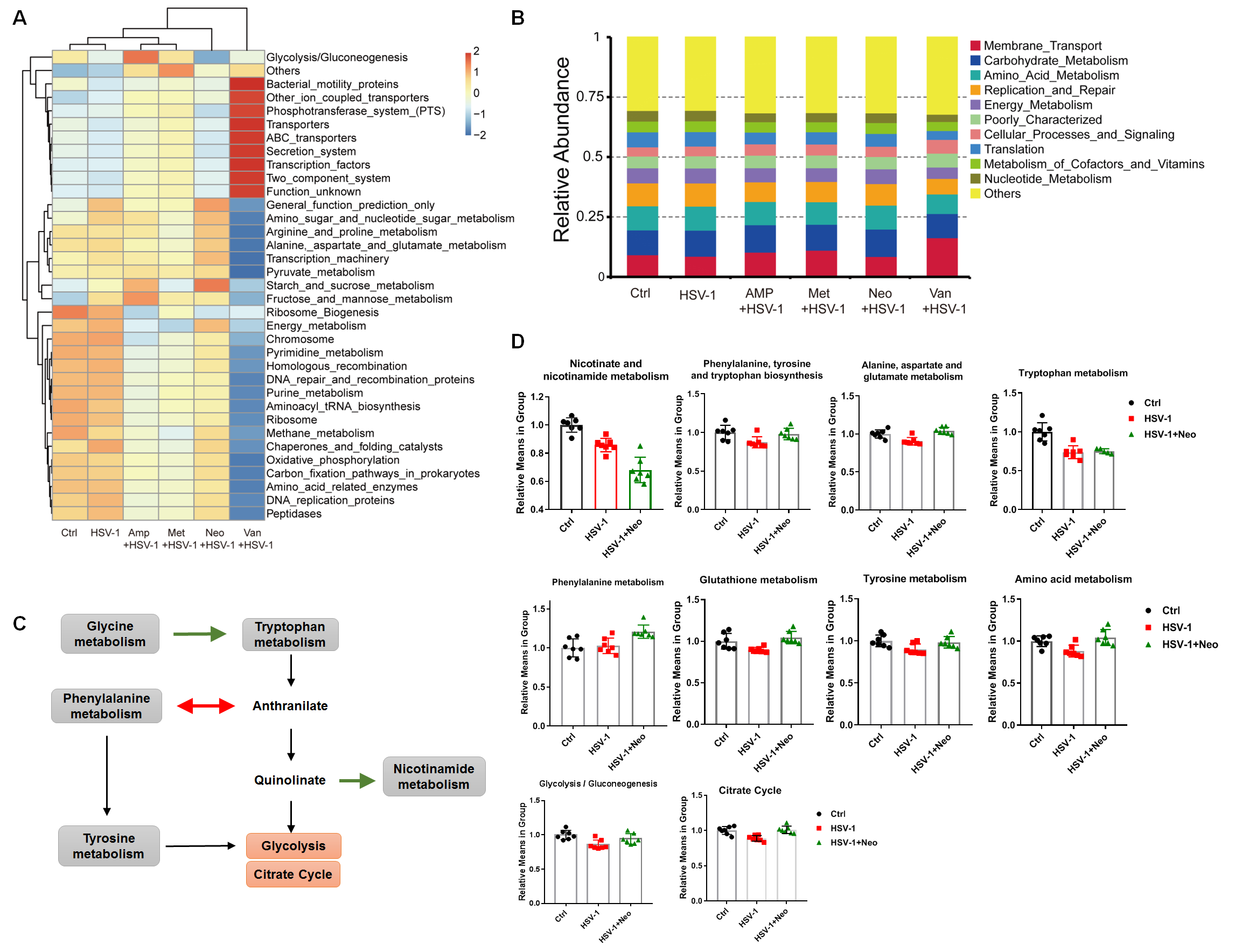
**

**Figure S14. Neomycin-sensitive bacteria regulates NAM and NAMO metabolism to restrict HSE. (A)** Functional analysis of differential regulated microbes in faecal samples from the Ctrl, HSV-1, HSV-1 + Neo, HSV-1 + Met, HSV-1 + Van, and HSV-1 + Amp mice. Red indicates upregulation and blue indicates downregulation, as shown in the scale bar. (**B**) PICRUSt functional enrichment analysis of differential regulated microbes in feces of Ctrl, HSV-1, HSV-1 + Neo, HSV-1 + Metro, HSV-1 + Vanco, and HSV-1 + Amp mice. **(C)** A schematic diagram depicting the interactions among different amino acid metabolisms. Red arrow indicates neomycin-mediated upregulation and green arrow indicates neomycin-mediated downregulation when compare with HSV-1 group. **(D)** Relative means of differential regulated microbes involved in different amino acid metabolisms in faecal samples from the Ctrl, HSV-1 and HSV-1 + Neo mice. Data are mean±SD**.** n=6-8 mice per group. Significance set as *p < 0.05, **p < 0.01, ***p < 0.001 versus HSV-1 group.
